# Association of lncRNA MEG3 rs941576 polymorphism, expression profile, and its related targets with the risk of obesity-related colorectal cancer: potential clinical insights

**DOI:** 10.1038/s41598-024-60265-6

**Published:** 2024-05-04

**Authors:** Mahmoud A. Senousy, Olfat G. Shaker, Ghada Ayeldeen, Abdullah F. Radwan

**Affiliations:** 1 Department of Biochemistry, Faculty of Pharmacy, Cairo University, Cairo, 11562 Egypt; 2 Department of Medical Biochemistry and Molecular Biology, Faculty of Medicine, Cairo University, Cairo, 11562 Egypt; 3 Department of Biochemistry and Molecular Biology, Faculty of Pharmacy, Egyptian Russian University, Cairo, 11829 Egypt

**Keywords:** CRC, Long non-coding RNA, microRNA, Obesity-related CRC, SNP, Cancer, Genetics, Molecular biology, Biomarkers, Diseases, Oncology, Risk factors

## Abstract

The identification of novel screening tools is imperative to empower the early detection of colorectal cancer (CRC). The influence of the long non-coding RNA maternally expressed gene 3 (MEG3) rs941576 single nucleotide polymorphism on CRC susceptibility remains uninvestigated. This research appraised MEG3 rs941576 association with the risk and clinical features of CRC and obesity-related CRC and its impact on serum MEG3 expression and its targets miR-27a/insulin-like growth factor 1 (IGF1)/IGF binding protein 3 (IGFBP3) and miR-181a/sirtuin 1 (SIRT1), along with the potential of these markers in obesity-related CRC diagnosis. 130 CRC patients (60 non-obese and 70 obese) and 120 cancer-free controls (64 non-obese and 56 obese) were enrolled. MEG3 targets were selected using bioinformatics analysis. MEG3 rs941576 was associated with magnified CRC risk in overall (OR (95% CI) 4.69(1.51–14.57), *P* = 0.0018) and stratified age and gender groups, but not with obesity-related CRC risk or MEG3/downstream targets’ expression. Escalated miR-27a and IGFBP3 and reduced IGF1 serum levels were concomitant with MEG3 downregulation in overall CRC patients versus controls and obese versus non-obese CRC patients. Serum miR-181a and SIRT1 were upregulated in CRC patients versus controls but weren’t altered in the obese versus non-obese comparison. Serum miR-181a and miR-27a were superior in overall and obesity-related CRC diagnosis, respectively; meanwhile, IGF1 was superior in distinguishing obese from non-obese CRC patients. Only serum miR-27a was associated with obesity-related CRC risk in multivariate logistic analysis. Among overall CRC patients, MEG3 rs941576 was associated with lymph node (LN) metastasis and tumor stage, serum MEG3 was negatively correlated with tumor stage, while SIRT1 was correlated with the anatomical site. Significant correlations were recorded between MEG3 and anatomical site, SIRT1 and tumor stage, and miR-27a/IGFBP3 and LN metastasis among obese CRC patients, while IGF1 was correlated with tumor stage and LN metastasis among non-obese CRC patients. Conclusively, this study advocates MEG3 rs941576 as a novel genetic marker of CRC susceptibility and prognosis. Our findings accentuate circulating MEG3/miR-27a/IGF1/IGFBP3, especially miR-27a as valuable markers for the early detection of obesity-related CRC. This axis along with SIRT1 could benefit obesity-related CRC prognosis.

## Introduction

Colorectal cancer (CRC) is the third most often recognized cancer in both genders and it ranks second in cancer-related deaths globally; it is the leading reason of death in men younger than 50 years^1^. According to GLOBOCAN 2020, colon cancer is regarded as the ninth most frequently occurring cancer in Egypt, whereas rectal cancer ranks 18th^2^. Egypt manifests a high rate of early CRC (under the age of 40) reaching 35% of CRC patients and often recognized at advanced stages; these patients display a dramatic drop in the 5-year survival rate and have poor prognosis^3,4^. This necessitates the development and implementation of CRC screening programs for preventive care.

More than 50% of all CRC cases and deaths are attributable to modifiable risk factors, including smoking, an unhealthy diet, high alcohol consumption, physical inactivity, and obesity^5^. In recent decades, there has been a rapid increment in the incidence of obesity and CRC^6^. Obesity, especially abdominal obesity, represents one of the chief environmental risk factors contributing to the incidence of CRC^6,7^, particularly in the Middle East^8^. Indeed, compelling evidence unveiled the relation between the early onset of obesity and a higher incidence of CRC, while weight loss and physical activity have been linked to a reduced CRC incidence^9^. However, the precise molecular underpinnings of the obesity-CRC association remain unclear. Besides, there are remarkable unmet medical needs and insufficient screening tools for obesity-related CRC. Therefore, comprehending the molecular basis of this association becomes a sobering thought that will evolve novel targets for the theragnostics of obesity-related CRC.

The pathogenesis of obesity-related CRC is composite and multifactorial. Obesity induces multiple mechanisms that instigate and advance the complex metabolic dysregulation of CRC tumorigenesis. These mechanisms include abnormal lipid metabolism, adipokines, hormones particularly insulin/insulin-like growth factor (IGF) and IGF binding proteins (IGFBPs), metabolic reprogramming, chronic low-grade inflammation, disrupted bile acid homeostasis, oxidative stress, neovascularization, and gut microbiota dysbiosis^6,9^. A potential influence has been shown for single nucleotide polymorphisms (SNPs) located in obesity-related genes as well as genome-wide association studies (GWAS) variants on the heightened risk of developing CRC^10,11^. In addition, the impact of DNA/RNA methylation, long non-coding RNAs (lncRNAs), and microRNAs (miRNAs)-based epigenetic mechanisms on the interconnection between obesity and the development and theragnostics of CRC was also spotlighted^12^. Interestingly, integrated transcriptome analysis of human visceral adipocytes uncovered the lncRNA:miRNA:mRNA networks of particular interest in obesity and CRC^13^.

The lncRNA maternally expressed gene 3 (MEG3) located on chromosome 14q32.3 is enacted as a tumor suppressor in multiple malignancies, including CRC^14,15^. Genetic variants of MEG3 have been repeatedly connected to cell phenotypes, escalated cancer risk, and toxicity of chemotherapy in various cancers^16–19^. Peculiarly, MEG3 rs7158663 SNP has been reportedly demonstrated as a useful predictive genetic marker for the risk of multiple cancers, including CRC^16–18^. Another MEG3 SNP rs941576, discovered in the imprinted region of chromosome 14q32.2, has been associated with disease-free survival in Chinese breast cancer women^19^. However, the impact of MEG3 rs941576 on CRC susceptibility and its association with the clinical features and risk factors of CRC, including obesity are yet unexplored.

The competitive endogenous RNA approach is one of the concrete regulatory mechanisms reported for lncRNAs via targeting various miRNAs/mRNAs axes^20,21^. Indeed, MEG3 overexpression inhibits CRC tumorigenesis, proliferation, and migration through sponging miR-141, miR-376, and miR-31^21–23^. Oncogenic miRNAs such as miR-27a and miR-181a were also identified as potential targets for MEG3^24–26^; in particular, these miRNAs have been extensively linked to obesity and obesity-related diseases^27^.

miR-27a is a well-known miRNA linked to lipid metabolism, metabolic reprogramming, adipocyte apoptosis, and macrophage activation. It plays a crucial role in the insulin signaling pathways, insulin resistance, and glucose metabolism through multiple targets, including IGF1^28–32^. miR-181a regulates the adipogenic process by targeting tumor necrosis factor-α^33^. miR-181a also targets sirtuin 1 (SIRT1)^34,35^, a NAD^+^-dependent deacetylase and a key regulator of cell growth and survival, metabolic reprogramming, and insulin sensitivity^29,34^. Interestingly, the miR-181 family members were among the differentially expressed miRNAs in obese CRC patients^13^. However, there is a paucity of literature about the clinical relevance of the MEG3/miR-27a/IGF1 and MEG3/miR-181a/SIRT1 co-expression networks in predicting obesity-related CRC.

This scenario prompted us to embark on this in-depth study with the aim of appraising the impact of MEG3 rs941576 SNP as a novel genetic marker of the risk and clinicopathological features of CRC as well as obesity-related CRC. The study also aimed to assess the association of this SNP with the expression of serum MEG3 and its downstream target networks miR-27a/IGF1/IGFBP3 and miR-181a/SIRT1 in CRC. In addition, the diagnostic and predictive abilities of these markers in obesity-related CRC patients were attested with the ultimate goal of enhancing the understanding of obesity-related CRC for improved screening and clinical management.

## Subjects and methods

### Patients

This case–control study included 250 adult individuals who were grouped as 130 CRC cases, the majority of whom had adenocarcinoma, and 120 cancer-free controls. All participants were enrolled from the Kasr Al-Ainy Hospital Gastrointestinal Endoscopy Unit, Cairo University. The diagnosis of CRC in the recruited personnel was confirmed by colonoscopy and positive pathology results.

At registration, complete medical history, physical examination, complete blood count, erythrocyte sedimentation rate (ESR), liver function tests, and fecal occult blood test were meticulously compiled for every participant. Epidemiological data and anthropometric parameters were collected through in-person interviews. The clinicopathological characteristics of CRC patients such as tumor staging as well as lymph node (LN) and distant metastasis were gathered and recorded in the medical records.

Inclusion criteria were adult patients (older than 18 years) of both sexes with confirmed CRC diagnosis. Patients with inflammatory bowel disease (IBD), another cancer, or prior therapy for CRC were excluded.

Healthy cancer-free subjects who were attending the same hospital and had the same age and gender as the patient population were regarded as the control group. The enrolled controls showed negative colonoscopy results for malignancy, polyps, and IBD.

Body mass index (BMI) was estimated to assess the obesity status in CRC cases and healthy controls using the weight (kg)/height (m^2^) formula. Both cases and controls were then subdivided according to their BMI into the overweight/obese group (BMI of at least 25 kg/m^2^) and the average weight (non-obese) group (BMI < 25 kg/m^2^)^36^. For simplification, the two groups were named obese and non-obese in this study.

The entire investigations and experiments were conducted per the ethical guidelines. A formal informed consent form has been signed by all participants or their legal representatives. The Ethics Committee of the Faculty of Pharmacy, Cairo University approved the study protocol and the informed consent (approval number BC3129) following the Helsinki Declaration's ethical principles.

### Blood samples collection

Six milliliters of blood were withdrawn from each participant. Three mL of blood were drawn into EDTA vacutainers for DNA extraction, while the remaining blood was collected into yellow gel vacutainers for serum separation. Within 30 min, the yellow vacutainers were centrifuged at 4000 rpm for 15 min to separate the serum from the clot. Aliquots of sera were separated and utilized for RNA extraction and protein assays of IGF1, IGFBP3, and SIRT1. All aliquots were stored at -80 degrees Celsius until use.

### SNP selection and genotyping

The NCBI dbSNP database was employed to select potential SNPs in the MEG3 gene. Seven SNPs were reported in the literature (rs7158663, rs10132552, rs11160608, rs4081134, rs7158663, rs3087918, and rs941576). The following selection criteria were implemented: a global minor allele frequency (MAF) greater than 10% reported in HapMap, reported in a prior GWAS study, has a recorded association with cancer risk, and previously studied in the Egyptian population. rs941576 was the only SNP previously linked to type 1 diabetes risk in a GWAS study^37^ and breast cancer risk in a population study^19^, and has been associated with the risk of acute ischemic stroke and rheumatoid arthritis in Egyptian patients^38,39^. Thus, this SNP has been selected for this study. To note, rs7158663 was extensively studied in cancer^16–18^, whereas rs10132552 and rs11160608 were not associated with cancer risk in some studies^40,41^. Although rs4081134 and rs3087918 have been associated with cancer risk^42,43^, to our knowledge, there is no published information about these variants in the Egyptian population.

Genomic DNA was extracted from whole EDTA-blood samples of all participants using the QIAamp DNA Mini Kit according to the manufacturer's guide (Qiagen, Valencia, CA). The DNA integrity was evaluated using the NanoDrop 2000c model (Thermo Scientific, USA). SNP genotyping was attempted using the real-time PCR TaqMan allelic discrimination assay. Amplification of DNA was conducted as previously described^20,44^ using the Qiagen Rotor-Gene Q Real-time PCR System by utilizing a TaqMan Master Mix and pre-designed primer/probe sets for rs941576 (A/G) [Catalog number: 4351379] (Thermo Scientific, USA). The PCR thermal cycler conditions (95 °C for ten minutes, then 40 cycles of 92 °C for fifteen s and 60 °C for ninety seconds) were implemented.

### Bioinformatics analysis to select lncRNA:miRNA:target gene co-expression networks

MEG3 was one of 8-top causal non-coding RNAs with strong evidence of being associated with colorectal adenocarcinoma in the LncRNA and Disease Database (LncRNADisease v3.0, http://www.rnanut.net/lncrnadisease/) (Supplementary Fig. S1). According to this database, the MEG3 score was 0.731059 with known causality and experimentally-validated link to colorectal adenocarcinoma. The mechanistic links to CRC were experimentally evidenced in both CRC cell lines^21–23^ and tumor tissues^45^.

Then we proceeded to collect the miRNA targets of MEG3 using the transcriptome-wide miRNA target predictions from the miRcode 11 database (http://www.mircode.org/). Interestingly, miR-181abcd/4262 and miR-27abc/27a-3p families were highly conserved targets of MEG3 according to this database. Then the interactions of MEG3 specifically with miR-27a and miR-181a have been checked to be experimentally verified in previous studies^24–26^.

The relationship of these selected miRNAs with colorectal neoplasms was verified in the Human MicroRNA Disease Database v4 (https://www.cuilab.cn/hmdd). The TargetScan 7.2 database (https://www.targetscan.org/vert_72/) was used for target predictions of miR-27a-3p and miR-181a-5p. IGF1 and SIRT1 were biologically relevant targets of miR-27a-3p and miR-181a-5p, respectively linked to metabolic reprogramming and glucose metabolism in cancer. The miRcode 11 database was employed to verify the interaction between miR-27a/IGF1 and miR-181a/SIRT1, which were also experimentally validated in cell lines^31,34,35^. The Pathway Studio online software was used to construct and visualize the interactions of MEG3 with the selected downstream targets with each other and with glucose.

### Assessment of serum MEG3, miR-27a, and miR-181a using reverse transcriptase-quantitative polymerase chain reaction (RT-qPCR)

A total of two hundred μL hemolysis-free serum was utilized to extricate the total RNA using QIAzol lysis reagent and the components of miRNeasy extraction kit provided by Qiagen, Valencia, CA. The NanoDrop 2000c model (Thermo scientific, USA) was employed to assess RNA concentration and purity. RNA samples with concentration ≥ 100 ng/mL and purity A260/A280 at least 1.8 were used in the cDNA synthesis for the selected lncRNA and miRNAs.

Total RNA (0.1 μg) was used in the cDNA synthesis of MEG3 in a final volume of twenty μL reverse transcription reactions by implementing the instructions of the RT^2^ first strand kit (Qiagen, Valencia, CA). The reverse transcription runs were attempted using appropriate thermal cycler conditions (10 min at 25 °C, 110 min at 37 °C, and 5 s at 95 °C). Before real-time PCR, the reverse transcription products were appropriately diluted with RNAase-free water.

The expression profile of MEG3 in the sera of cases and controls was examined by employing the housekeeping gene glyceraldehyde 3-phosphate dehydrogenase (GAPDH) as an internal control for normalization. Specific custom-made primers (Invitrogen) predesigned using primer3web software (https://primer3.ut.ee/) were used. Before customization, the specificity of primers was validated using the NCBI primer-blast tool (https://www.ncbi.nlm.nih.gov/tools/primer-blast/). ﻿Primer sequences are mentioned in Table 1. In short, qPCR was carried out on the Qiagen Rotor-Gene Q System using twenty μL reaction mixtures prepared using the PCR Maxima SYBR Green kit (Thermo Scientific, USA) along with forward and reverse primers as previously described^20,44^. The thermal cycler runs were ten minutes at 95 °C, followed by 45 cycles of fifteen seconds at 95 °C and sixty seconds at 60 °C.

**Table 1:** Customized primer sequences used in the study.

Gene	Primer sequence	
MEG3	Forward	5′- CTGCCCATCTACACCTCACG-3′
	Reverse	5′- CTCTCCGCCGTCTGCGCTAGGGGCT-3′
GAPDH	Forward	5′-CCCTTCATTGACCTCAACTA-3′
	Reverse	5′- TGGAAGATGGTGATGGGATT-3′

*MEG3* maternally expressed gene 3, *GAPDH* glyceraldehyde 3-phossphate dehydrogenase.

For miRNAs, the miScript II RT Kit provided by Qiagen was employed to perform reverse transcription as directed by the manufacturer using total RNA (0.1 μg) in twenty μL reactions. The cDNA samples were appropriately diluted and amplified using the miScript SYBR Green PCR kit (Qiagen) and the supplied miScript Universal Primer (reverse primer), along with ready-made specific miScript Primer (forward primer) Assays for hsa-miR-27a-3p, hsa-miR-181a-5p, and the housekeeping miScript PCR control miRNA SNORD68 (Qiagen). SNORD68 was validated as an internal control for normalization of miRNAs in several studies which supported its use as a reference for miRNA relative quantification based on its stable and equivalent expression between the sera of diseased patients and controls^20,44,46^. Briefly, real-time PCR was performed in 20 μL reaction mixtures prepared as previously described^20,46^ using the Qiagen Rotor-Gene Q system. Thermal conditions of 95 °C for thirty minutes, followed by 40 cycles at 94 °C for fifteen seconds, 55 °C for thirty seconds, and 70 °C for thirty seconds were implemented for all PCR runs.

The PCR products’ specificity was checked using melting curve analysis. The expression of genes relative to internal control (2^−∆Ct^) was calculated. For relative quantification, the fold change was expressed using the 2^−∆∆Ct^ formula.

### Assessment of serum SIRT1, IGF1, and IGFBP3 using enzyme-linked immunosorbent assay (ELISA)

 The human SIRT1, IGF1, and IGFBP3 ELISA kits provided by Abcam (Trumpington, Cambridge, UK, catalog numbers ab171573, ab211651, and ab211652) were used for the quantitative assessment of SIRT1, IGF1, and IGFBP3 in the sera of studied participants as directed by the production company.

### Sample size calculation

At the beginning of the study, the sample size was estimated using the G*Power software version 3.1.9.7 by assuming the following: two independent groups (CRC cases versus controls), effect size = 0.5 (fold change 1.25 in cases versus 1 in control), population variance (SD = 0.5), case/control ratio = 1, type I error α = 0.05, and type II error β = 0.2. A minimum total sample size of 128 yielded a two-tailed power (1 − β) = 0.8, while a total sample size of 250 (130 + 120) yielded a two-tailed power reaching 97% based on these assumptions.

For SNP analysis, this calculated sample size was checked using the web-based Power Calculator for Genetic Studies (http://www.sph.umich.edu/csg/abecasis/CaTS/index.html). The following postulations were fed to the power calculator: significance level α = 0.05, a multiplicative model, a predicted risk allele frequency of ≥ 0.25, odds ratio (OR) of ≥ 1.7, and disease prevalence in the adult Egyptian population as previously reported^47^. From the power calculation, a minimum sample size of 130 cases and 120 controls yielded 80% power.

### Statistical methods

Data were analyzed using GraphPad Prism 9.5.1 statistics program (CA, USA). Categorical data were expressed as numbers and percentages, whereas numerical data were presented using mean ± standard deviation, median (25%-75% percentiles), or range when applicable. Using Kolmogorov–Smirnov, D'Agostino & Pearson, and Shapiro–Wilk tests, the data normality was determined. The student's t-test, Mann–Whitney U test, one-way ANOVA followed by Tukey’s, or Kruskal–Wallis followed by Dunn’s post-hoc test were used to compare numerical variables when applicable. To compare categorical data, Fisher's exact test was employed. The online SNPStats online tool (https://snpstats.net/) was utilized to conduct the SNP analysis. The investigated SNP was screened for Hardy–Weinberg equilibrium (HWE) departure using a chi-square test. Unconditional logistic regression models, odds ratios (OR), and 95% confidence intervals (CI) were used to assess the associations of the tested SNP with CRC risk or the clinicopathological data adjusted with confounders. The diagnostic accuracy of the tested markers was evaluated using receiver-operating characteristic (ROC) analysis and the area under the curve (AUC) was computed. AUCs were classified into three categories: AUC = 0.6 to < 0.7, 0.7 to < 0.9, and ≥ 0.9 to designate the marker as a significant, promising, and excellent discriminator, respectively. To categorize the predictor variables associated with the risk of obesity-related CRC in obese controls, the univariate and multivariate logistic regression analyses were performed. A stepwise-forward multivariate analysis was computed using relevant significant variables from the univariate analysis to estimate the final variables associated with the likelihood of being diagnosed with obesity-related CRC. The correlations between the measurements were evaluated using Spearman's rho correlation coefficient. The results were considered statistically significant when the two-tailed *P*-value of the test was less than 0.05.

### Ethics approval

A formal informed consent form has been signed by all participants or their legal representatives. The Ethics Committee of the Faculty of Pharmacy, Cairo University approved the study protocol and the informed consent (approval number BC3129) following the Helsinki Declaration's ethical principles.

## Results

### The demographic, laboratory, and clinical features of CRC and healthy controls

The demographic, laboratory, and clinicopathological data of the tested groups are recorded in Table 2. More than 50% of CRC patients were < 50 years old. A male predominance was observed in CRC patients (64.6%) and smoking was observed as a risk factor in 30% of patients. CRC and controls were matched regarding the obesity status (*P* = 0.311), where 54.8% of CRC patients were overweight/obese versus 46.7% in healthy controls. Regarding the laboratory investigations, there was no significant difference between the studied groups regarding total leukocyte count (TLC) and platelet count; however, CRC patients showed a remarkable increase in the erythrocyte sedimentation rate (ESR) compared with that in the healthy control group (*P* = 0.001).

**Table 2:** Demographic, laboratory, and clinicopathological data of the studied groups.

Parameter	Healthy controls (n = 120)	CRC (n = 130)	*P-*value
Age (years), n (%)			
< 50	72 (60)	66 (50.8)	0.162
≥ 50	48 (40)	64 (49.2)	
Age range	25–76	23–70	
Sex, n (%)			
Male	76 (63.3)	84 (64.6)	0.893
Female	44 (36.7)	46 (35.4)	
Obesity, n (%)			
Non-obese (average weight)	64 (53.3)	60 (46.2)	0.311
Overweight/obese	56 (46.7)	70 (54.8)	
Smoking, n (%)			
Non-smokers	95 (79.2)	91 (70)	0.111
Smokers	25 (20.8)	39 (30)	
Platelet count × 10^3^/mm^3^	248.6 ± 39.98	276.6 ± 94.48	0.156
TLC × 10^3^/mm^3^	6.44 ± 1.33	6.98 ± 2.63	0.071
ESR (mm/h)	20.1 ± 11	46 ± 31.38	**0.001**
Tissue type			
Adenocarcinoma		120 (92.3)	
Non-adenocarcinoma		10 (7.7)	
Anatomical site, n (%)			
Colon		79 (60.7)	
Rectum		51 (39.3)	
Tumor stage, n (%)			
Stages I–II		86 (66)	
Stages III–IV		44 (34)	
LN metastasis, n (%)			
Present		60 (46.2)	
Absent		70 (53.8)	
Distant metastasis, n (%)			
Present		20 (15.4)	
Absent		110 (84.6)	

Data of the studied groups are expressed as mean ± SD or number (percentage). Fischer’s exact test was employed to compare categorical data. Numerical data were compared using the t-test. *P* < 0.05 (bold) is statistically significant.

*CRC* colorectal cancer, *ESR* erythrocyte sedimentation rate, *LN* lymph node, *TLC* total leukocyte count.

The clinicopathological data portrayed tumors with variable size (1.5 cm or greater) located in the colon more than the rectum (60.7% versus 39.3%). Overall, 92.3% of CRC tumors were adenocarcinoma. Among CRC patients, 46.2% presented with lymph node (LN) metastasis, while only 15.4% of the patients had metastatic CRC, with hepatic focal lesions being present in all. Furthermore, 66% of CRC patients have been diagnosed with the American Joint Committee on Cancer (AJCC) as early stages (I and II); however, 34% of the diagnosed CRC patients with late stages (III and IV).

### Association of MEG3 rs941576 (A/G) SNP with the risk of CRC

Referring to Ensembl release 111—Jan 2024, MEG3 rs941576 (A/G) is described as an intron variant and a non-coding RNA transcript variant; its MAF in the control group (G = 0.12) was lower than the global MAF (G = 0.38) (Supplementary Table S1). The distribution of the rs941576 genotypes in the control group didn’t stray significantly from Hardy-Weinberg equilibrium (HWE) (*P* = 0.067) (Supplementary Table S2).

Table 3 demonstrates the allele and genotype frequencies of MEG3 rs941576 in healthy control and CRC patients. The distribution of the minor G allele in CRC patients was higher than that in the control group (20% versus 12%) implying a significant risk of CRC [G vs A, adjusted OR = 1.64, *P* = 0.019]. The rs941576 minor GG genotype was significantly associated with a 4.65- and 4.69-fold escalating risk of CRC in the codominant and recessive models, respectively [GG vs AA, adjusted OR = 4.65, *P* = 0.011 and GG vs AA + AG, adjusted OR = 4.69, *P* = 0.0018, respectively] adjusted with age, sex, and obesity as confounders. When applying a stringent significance level to correct for multiple comparisons (Bonferroni *P* < 0.01), the results are still significant in the recessive model (GG vs AA + AG).

**Table 3:** Association of MEG3 rs941576 (A/G) SNP with the risk of CRC.

Model	Genotype	Frequency in control group (n = 120)	Frequency in CRC group (n = 130)	Adjusted OR (95% CI)	*P*^a^-value	AIC	BIC
Codominant	AA	95 (79.2%)	94 (72.3%)	1.00	**0.011**	345.5	366.6
	AG	21 (17.5%)	19 (14.6%)	0.95 (0.47–1.91)			
	GG	4 (3.3%)	17 (13.1%)	**4.65 (1.49–14.52)**			
Dominant	AA	95 (79.2%)	94 (72.3%)	1.00	0.16	350.5	368.1
	AG + GG	25 (20.8%)	36 (27.7%)	1.54 (0.84–2.80)			
Recessive*	AA + AG	116 (96.7%)	113 (86.9%)	1.00	**0.0018**^**b**^	343.5	361.1
	GG	4 (3.3%)	17 (13.1%)	**4.69 (1.51–14.57)**			
Overdominant	AA + GG	99 (82.5%)	111 (85.4%)	1.00	0.57	352.1	369.7
	AG	21 (17.5%)	19 (14.6%)	0.82 (0.41–1.64)			
Allelic	A	211 (0.88)	207 (0.8)	1.00	**0.019**	347.2	364.8
	G	29 (0.12)	53 (0.2)	**1.64 (1.08–2.51)**			

Data were computed using the SNPStats online software.

*CI* confidence interval, *CRC* colorectal cancer, *OR* odds ratio.

*Indicates the best fit model based upon the lowest Akaike Information Criterion (AIC) and Bayesian Information Criterion (BIC).

^a^Adjusted with age, sex, and obesity in a logistic regression model.

^b^Significant after applying Bonferroni correction for multiple testing (*P* < 0.01). *P* < 0.05 (bold) is statistically significant.

### Risk stratification analysis of MEG3 rs941576 (A/G) SNP association with CRC by age, sex, and obesity

Subsequently, the association of MEG3 rs941576 with CRC risk in stratified groups according to sex, age, and obesity was assessed (Table 4). Compared to healthy controls, males harboring the minor GG genotype of this SNP had a significantly higher risk for CRC than females in the codominant and recessive models, while the combined AG + GG genotypes were associated with markedly increased risk of CRC in females in the dominant model. Interestingly, the AG genotype was protective in patients with age < 50 years, while was associated with an escalated risk of CRC in the older patients ≥ 50 years in the codominant and overdominant models. The combined AG + GG genotype was markedly associated with an elevated risk of CRC in the older patient group (≥ 50 years) in the dominant model. Conversely, this SNP didn’t show a significant association with CRC risk when patients and controls were classified into obese and non-obese.

**Table 4:** Cross-interaction of MEG3 rs941576 (A/G) with sex, age, and obesity as risk factors of CRC.

		Control (n = 44)	CRC (n = 46)	Adjusted OR^a^ (95% CI)	Control (n = 76)	CRC (n = 84)	Adjusted OR^a^ (95% CI)
rs941576 A/G and sex cross-classification interaction							
Model	Genotype	Females			Males		
Codominant	AA	43	34	1.00	52	60	1.52 (0.84–2.74)
	AG	1	6	7.07 (0.80–62.60)	20	13	0.92 (0.39–2.14)
	GG	0	6	–	4	11	**3.9 (1.09–13.94)**
	Interaction *P*-value: 0.014						
Dominant	AA	43	34	1.00	52	60	1.51 (0.84–2.72)
	AG + GG	1	12	**15.10 (1.84–124.04)**	24	24	1.38 (0.65–2.91)
	Interaction *P*-value: 0.0021						
Recessive	AA + AG	44	40	1.00	72	73	1.20 (0.70–2.09)
	GG	0	6	–	4	11	**3.64 (1.02–12.96)**
	Interaction *P*-value: 0.097						
Overdominant	AA + GG	43	40	1.00	56	71	1.43 (0.81–2.52)
	AG	1	6	6.03 (0.69–53.08)	20	13	0.78 (0.34–1.80)
	Interaction *P*-value: 0.018						

		Control (n = 72)	CRC (n = 66)	Adjusted OR^b^ (95% CI)	Control (n = 48)	CRC (n = 64)	Adjusted OR^b^ (95% CI)
rs941576 A/G and age cross-classification interaction							
Model	Genotype	Age < 50			Age ≥ 50		
Codominant	AA	48	54	1.00	47	40	0.75 (0.42–1.34)
	AG	20	6	**0.27 (0.10–0.72)**	1	13	**11.72 (1.47–93.64)**
	GG	4	6	1.42 (0.38–5.38)	0	11	–
	Interaction *P*-value: < 0.0001						
Dominant	AA	48	54	1.00	47	40	0.75 (0.42–1.34)
	AG + GG	24	12	0.45 (0.20–1.01)	1	24	**23.11 (2.99–178.78)**
	Interaction *P*-value: < 0.0001						
Recessive	AA + AG	68	60	1.00	48	53	1.25 (0.74–2.12)
	GG	4	6	1.81 (0.48–6.76)	0	11	–
	Interaction *P*-value: 0.013						
Overdominant	AA + GG	52	60	1.00	47	51	0.95 (0.55–1.63)
	AG	20	6	**0.25 (0.09–0.68)**	1	13	**11.04 (1.39–87.64)**
	Interaction *P*-value: < 0.0001						

		Control (n = 64)	CRC (n = 60)	Adjusted OR^c^ (95% CI)	Control (n = 56)	CRC (n = 70)	Adjusted OR^c^ (95% CI)
rs941576 A/G and obesity cross-classification interaction							
Model	Genotype	Non-obese			Obese		
Codominant	AA	48	40	1.00	47	54	1.40 (0.79–2.50)
	AG	12	9	0.88 (0.33–2.35)	9	10	1.38 (0.51–3.76)
	GG	4	11	2.93 (0.85–10.14)	0	6	–
	Interaction *P*-value: 0.25						
Dominant	AA	48	40	1.00	47	54	1.39 (0.78–2.47)
	AG + GG	16	20	1.46 (0.65–3.29)	9	16	2.26 (0.89–5.73)
	Interaction *P*-value: 0.86						
Recessive	AA + AG	60	49	1.00	56	64	1.43 (0.84–2.43)
	GG	4	11	3.02 (0.89–10.24)	0	6	–
	Interaction *P*-value: 0.097						
Overdominant	AA + GG	52	51	1.00	47	60	1.35 (0.78–2.35)
	AG	12	9	0.76 (0.29–2.01)	9	10	1.19 (0.44–3.20)
	Interaction *P*-value: 0.84						

Data were computed using the SNPStats online software.

^a^Adjusted with age and obesity.

^b^Adjusted with sex and obesity.

^c^Adjusted with age and sex in a logistic regression model. *P* < 0.05 (bold) is statistically significant.

*CI* confidence interval, *CRC* colorectal cancer, *OR* odds ratio.

### Association of MEG3 rs941576 (A/G) SNP with the clinicopathological characteristics of the overall CRC group

The association of MEG3 rs941576 SNP with the clinicopathological data of CRC patients was further examined (Table 5). We recorded a significant positive association of this SNP with LN metastasis (dominant model AG + GG vs AA, adjusted OR = 2.65, *P* = 0.024). Additionally, the frequency of the risk GG genotype was significantly higher in CRC patients with late tumor stages (III-IV) than those with early tumor stages (I-II) in the codominant (GG vs AA, adjusted OR = 3.34, *P* = 0.036) and recessive (GG vs AA + AG, adjusted OR = 3.16, *P* = 0.041) models. However, there was no significant association of this SNP with anatomical site (colon vs rectum) or distant metastasis (*P* > 0.05). All ORs and *P*-values were adjusted with age, sex, and obesity status. The full analysis results of the five genetic models are listed in Supplementary Tables S3–S6.

**Table 5:** Association of MEG3 rs941576 SNP with the clinicopathological data of CRC patients.

Model	Genotype	Colon (n = 79)	Rectum (n = 51)	Adjusted OR^a^ (95% CI)	*P*^a^-value
Recessive	AA + AG	72 (91.1%)	41 (80.4%)	1.00	0.13
	GG	7 (8.9%)	10 (19.6%)	2.28 (0.77–6.80)	

Model	Genotype	No LN metastasis (n = 70)	LN-metastasis (n = 60)	Adjusted OR^a^ (95% CI)	*P*^a^-value
Dominant	AA	54 (77.1%)	40 (66.7%)	1.00	**0.024**
	AG + GG	16 (22.9%)	20 (33.3%)	**2.65 (1.11–6.31)**	

Model	Genotype	No distant metastasis (n = 110)	Distant metastasis (n = 20)	Adjusted OR^a^ (95% CI)	*P*^a^-value
Dominant	AA	78 (70.9%)	16 (80%)	1.00	0.43
	AG + GG	32 (29.1%)	4 (20%)	0.62 (0.19–2.09)	

Model	Genotype	Tumor stage I–II (n = 86)	Tumor stage III–IV (n = 44)	Adjusted OR^a^ (95% CI)	*P*^a^-value
Codominant	AA	66 (76.7%)	28 (63.6%)	1.00	**0.036**
	GG	8 (9.3%)	9 (20.4%)	**3.34 (1.07–10.38)**	
Recessive	AA + AG	78 (90.7%)	35 (79.5%)	1.00	**0.041**
	GG	8 (9.3%)	9 (20.4%)	**3.16 (1.04–9.61)**	

All the genetic models were tested using the SNPStats online software and the significant model is presented. In case of non-significant associations, the best fit model (based upon the lowest AIC and BIC) is presented.

^a^Adjusted for age, sex, and obesity in a logistic regression model. *P* < 0.05 (bold) is statistically significant.

*CI* confidence interval, *CRC* colorectal cancer, *LN* lymph node, *OR* odds ratio.

### Association of MEG3 rs941576 (A/G) SNP with the clinicopathological data among obese and non-obese CRC subgroups

We further scrutinized the MEG3 rs941576 SNP cross-interaction with the clinicopathological data among obese and non-obese CRC patients (Table 6). A trend of interaction was observed with distant metastasis in obese CRC patients in the dominant model (AG + GG vs AA, interaction *P* = 0.032). Interestingly, this SNP demonstrated an association with tumor stage in obese CRC patients in the recessive and overdominant models; however, the overall interaction *P*-values didn’t reach statistical significance (*P* > 0.05). There was no significant interaction of this SNP with the anatomical site (colon vs rectum) or LN metastasis (*P* > 0.05) relative to the obesity status in CRC patients. All ORs and *P*-values were adjusted with age and sex.

**Table 6:** Cross-interaction of MEG3 rs941576 (A/G) with clinicopathological data among obese and non-obese CRC patients.

		Non-obese (n = 37)	Obese (n = 42)	Adjusted OR^a^ (95% CI)	Non-obese (n = 23)	Obese (n = 28)	Adjusted OR^a^ (95% CI)
rs941576 A/G and anatomical site cross-classification interaction							
Model	Genotype	Colon			Rectum		
Codominant	AA	28	34	1.00	12	20	1.14 (0.45–2.93)
	AG	6	4	0.44 (0.10–1.92)	3	6	2.63 (0.54–12.77)
	GG	3	4	0.79 (0.15–4.21)	8	2	0.24 (0.04–1.41)
	Interaction *P*-value: 0.13						
Dominant	AA	28	34	1.00	12	20	1.13 (0.44–2.89)
	AG + GG	9	8	0.56 (0.18–1.81)	11	8	0.82 (0.26–2.62)
	Interaction *P*-value: 0.77						
Recessive	AA + AG	34	38	1.00	15	26	1.54 (0.66–3.59)
	GG	3	4	0.90 (0.17–4.68)	8	2	0.27 (0.05–1.51)
	Interaction *P*-value: 0.17						
Overdominant	AA + GG	31	38	1.00	20	22	0.86 (0.37–2.00)
	AG	6	4	0.44 (0.10–1.94)	3	6	2.83 (0.59–13.64)
	Interaction *P*-value: 0.066						

		Non-obese (n = 38)	Obese (n = 32)	Adjusted OR^a^ (95% CI)	Non-obese (n = 22)	Obese (n = 38)	Adjusted OR^a^ (95% CI)
rs941576 A/G and LN metastasis cross-classification interaction							
Model	Genotype	No LN metastasis			LN metastasis		
Codominant	AA	26	28	1.00	14	26	1.78 (0.72–4.39)
	AG	6	2	0.37 (0.06–2.22)	3	8	2.72 (0.61–12.12)
	GG	6	2	0.35 (0.06–2.03)	5	4	0.65 (0.14–2.98)
	Interaction *P*-value: 0.46						
Dominant	AA	26	28	1.00	14	26	1.77 (0.72–4.34)
	AG + GG	12	4	0.36 (0.10–1.36)	8	12	1.40 (0.46–4.20)
	Interaction *P*-value: 0.37						
Recessive	AA + AG	32	30	1.00	17	34	2.15 (0.94–4.92)
	GG	6	2	0.40 (0.07–2.27)	5	4	0.73 (0.16–3.33)
	Interaction p-value: 0.89						
Overdominant	AA + GG	32	30	1.00	19	30	1.64 (0.73–3.69)
	AG	6	2	0.44 (0.07–2.61)	3	8	3.08 (0.70–13.57)
	Interaction *P*-value: 0.2						

		Non-obese (n = 54)	Obese (n = 56)	Adjusted OR^a^ (95% CI)	Non-obese (n = 6)	Obese (n = 14)	Adjusted OR^a^ (95% CI)
rs941576 A/G and distant metastasis cross-classification interaction							
Model	Genotype	No distant metastasis			Distant metastasis		
Codominant	AA	34	44	1.00	6	10	1.36 (0.42–4.35)
	AG	9	8	0.75 (0.24–2.32)	0	2	–-
	GG	11	4	0.32 (0.09–1.20)	0	2	–-
	Interaction *P*-value: 0.12						
Dominant	AA	34	44	1.00	6	10	1.36 (0.42–4.36)
	AG + GG	20	12	0.52 (0.21–1.29)	0	4	–-
	Interaction *P*-value: **0.032**						
Recessive	AA + AG	43	52	1.00	6	12	1.87 (0.62–5.69)
	GG	11	4	0.34 (0.09–1.23)	0	2	–-
	Interaction *P*-value: 0.25						
Overdominant	AA + GG	45	48	1.00	6	12	1.78 (0.58–5.48)
	AG	9	8	0.91 (0.30–2.78)	0	2	–-
	Interaction *P*-value: 0.11						

		Non-obese (n = 46)	Obese (n = 40)	Adjusted OR^a^ (95% CI)	Non-obese (n = 14)	Obese (n = 30)	Adjusted OR^a^ (95% CI)
Model	Genotype	Tumor stage I–II			Tumor stage III–IV		
Codominant	AA	32	34	1.00	8	20	2.49 (0.90–6.87)
	AG	8	4	0.51 (0.13–2.05)	1	6	8.92 (0.93–85.57)
	GG	6	2	0.40 (0.07–2.28)	5	4	0.68 (0.14–3.26)
	Interaction *P*-value: 0.27						
Dominant	AA	32	34	1.00	8	20	2.45 (0.89–6.73)
	AG + GG	14	6	0.46 (0.15–1.44)	6	10	1.87 (0.55–6.39)
	Interaction *P*-value: 0.58						
Recessive	AA + AG	40	38	1.00	9	26	**3.46 (1.35–8.88)**
	GG	6	2	0.44 (0.08–2.48)	5	4	0.76 (0.16–3.55)
	Interaction *P*-value: 0.57						
Overdominant	AA + GG	38	36	1.00	13	24	2.01 (0.83–4.84)
	AG	8	4	0.57 (0.14–2.26)	1	6	**10.13 (1.07–96.20)**
	Interaction p-value: 0.081						

Data were computed using the SNPStats online software.

^a^Adjusted with age and sex in a logistic regression model. *P* < 0.05 (bold) is statistically significant.

*CI* confidence interval, *CRC* colorectal cancer, *OR* odds ratio, *LN* lymph node.

### Serum MEG3 and its downstream targets are differentially expressed in CRC patients

**Figure 1:**
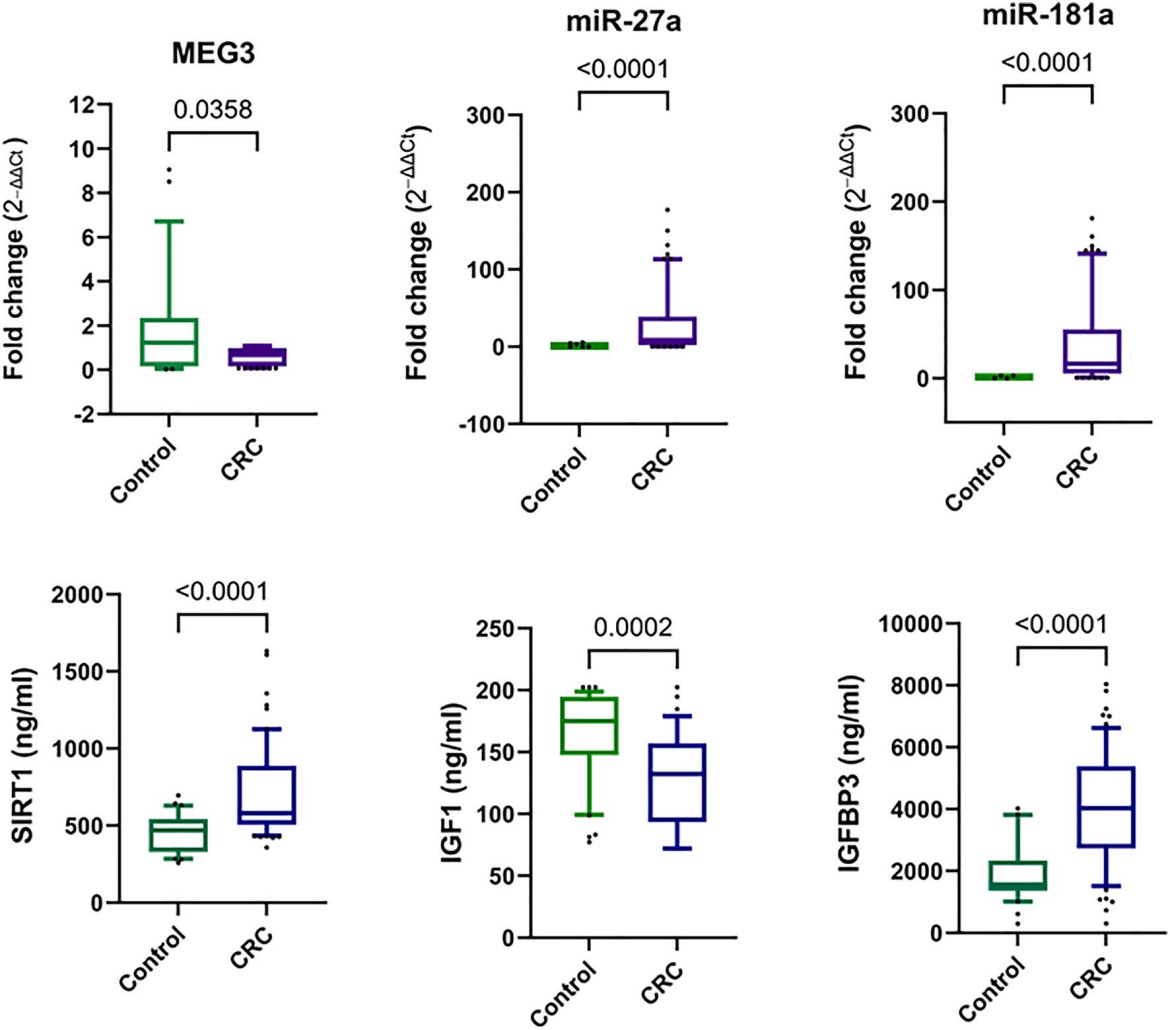
Differential expression of serum MEG3, miR-27a, miR-181a, SIRT1, and IGF1/IGFBP3 axis in CRC patients versus healthy controls. The box pinpoints the 25%-75% percentiles; the line inside the box pinpoints the median and the upper and lower lines represent the 10%-90% percentiles of studied parameters’ levels. The comparison between CRC (n = 130) and healthy controls (n = 120) was done using the non-parametric Mann–Whitney U test. Fold change was calculated using ΔΔCt method. *P* < 0.05 was set as the statistical significance level. *MEG3* maternally expressed gene 3, *IGF1* insulin-like growth factor 1, *IGFBP3* IGF binding protein 3, *SIRT1* sirtuin 1.

Compared with levels in the healthy control group, serum MEG3 expression levels were downregulated (median fold change = 0.675,* P* = 0.0358), whereas serum miR-27a and miR-181a expression levels showed marked upregulation reaching a median of 16.28-fold and 8.34-fold (*P* < 0.0001 for each), respectively in CRC patients (Fig. 1).

As depicted in Fig. 1, serum SIRT1 levels were upregulated in CRC patients compared with levels in the control group. The IGF1/IGFBP3 axis was differentially expressed in the sera of CRC patients compared with healthy controls, where levels of IGF1 showed a marked decrease (*P* = 0.0002), whereas levels of its major binding protein IFGBP3 were substantially increased (*P* < 0.0001) in comparison with their levels in the healthy control group.

### Association of MEG3 rs941576 (A/G) SNP with serum MEG3 expression levels and other parameters in CRC patients

**Figure 2:**
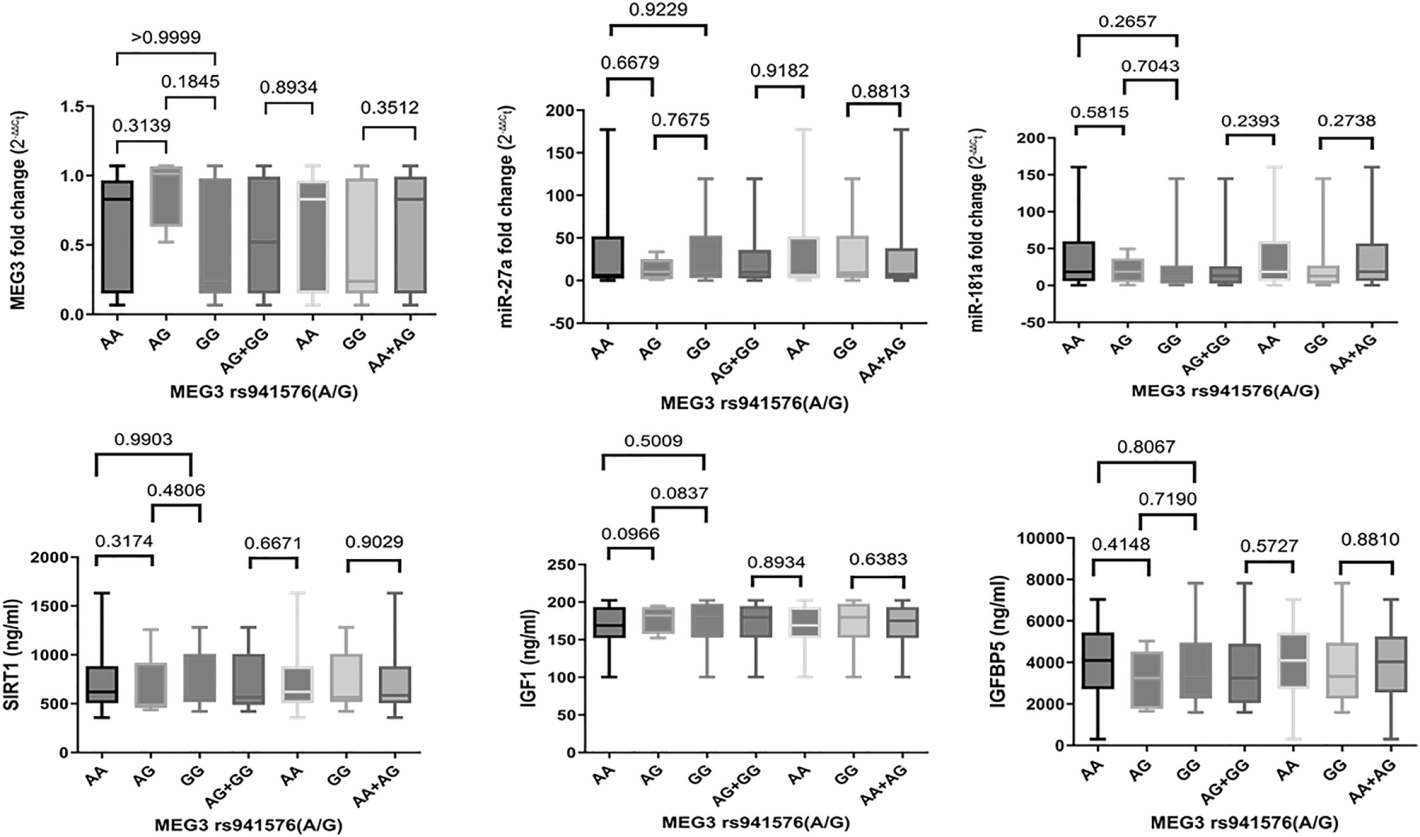
Association of MEG3 rs941576 (A/G) SNP with serum levels of the measured parameters in CRC patients. Data are presented as mean ± SD. Comparison of each parameter’s levels between CRC patients with different genotypes (AA = 94, AG = 19, and GG = 17) was done using the non-parametric Kruskal–Wallis test with the Dunn’s post-hoc test (codominant model) or the Mann–Whitney U test (dominant and recessive models). *P* < 0.05 was set as the statistical significance level. *MEG3* maternally expressed gene 3, *IGF1* insulin-like growth factor 1, *IGFBP3* IGF binding protein 3, *SIRT1* sirtuin 1.

To further elucidate the possible role of MEG3 rs941576 SNP in predisposing CRC, the mechanistic impact of MEG3 rs941576 SNP on MEG3 expression was contemplated; however, we failed to find a significant association of rs941576 genotypes with serum MEG3 expression levels in the recruited CRC patients in the codominant, dominant, and recessive models (*P* > 0.05) (Fig. 2). Likewise, this SNP was not significantly associated with serum miR-27a, miR-181a, SIRT1, IGF1, and IGFBP3 levels in these models among the studied CRC patients (*P* > 0.05) (Fig. 2).

### Serum MEG3/miR-27a/IGF1/IGFBP3 axis is differentially expressed in obese CRC versus non-obese CRC patients

**Figure 3:**
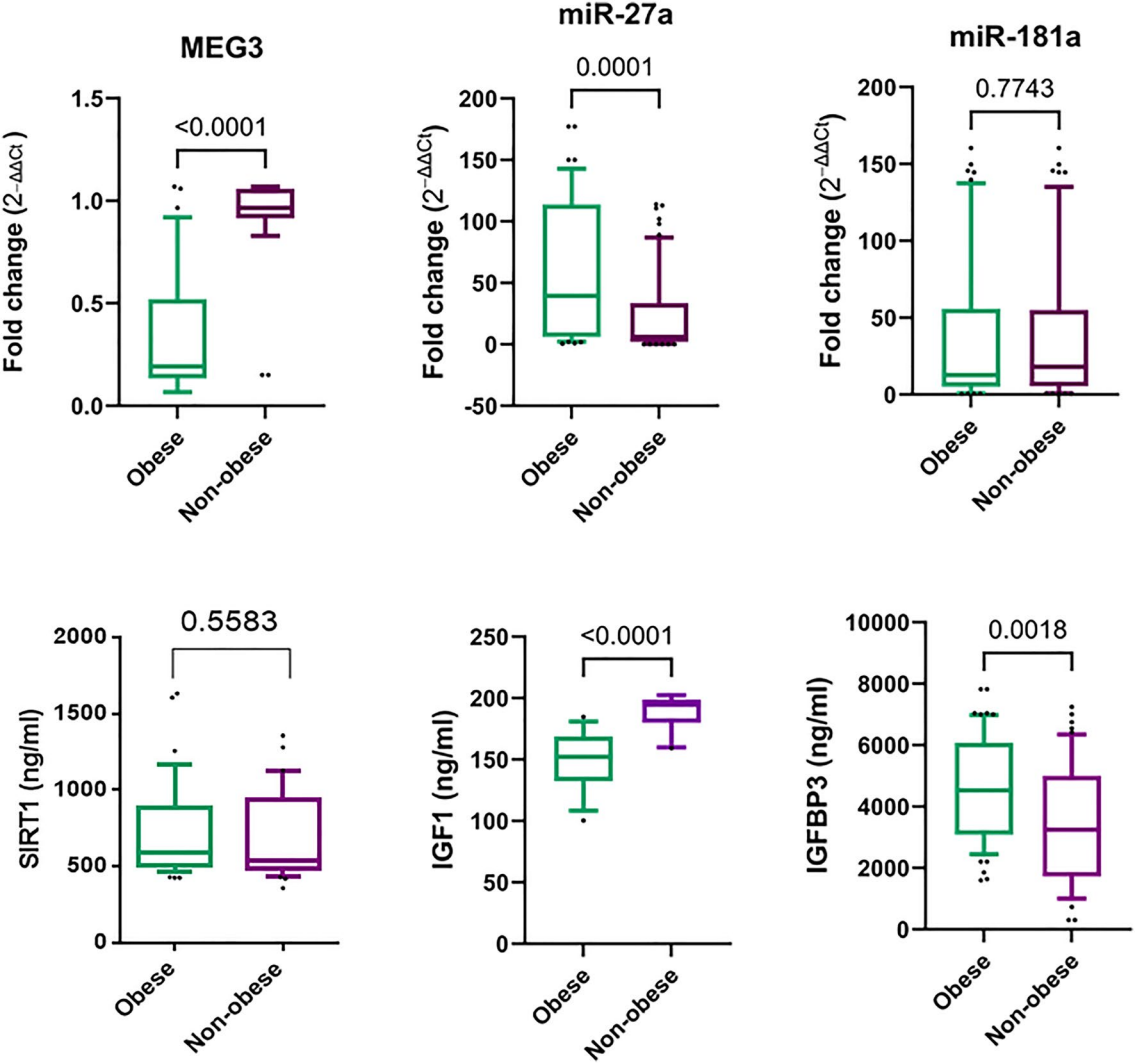
Differential expression of MEG3/miR-27a/IGF1/IGFBP3 axis in obese versus non-obese CRC patients. The box pinpoints the 25%-75% percentiles; the line inside the box pinpoints the median and the upper and lower lines represent the 10–90% percentiles of studied parameters’ levels. The comparison between obese CRC (n = 70) and non-obese CRC (n = 60) patients was done using the non-parametric Mann–Whitney U test. Fold change was calculated using ΔΔCt method with normalization of the obese CRC data against obese controls (n = 56) and the non-obese CRC data against their corresponding controls (n = 64). *P* < 0.05 was set as the statistical significance level. *MEG3* maternally expressed gene 3, *IGF1* insulin-like growth factor 1, *IGFBP3* IGF binding protein 3.

When it comes to comparing serum levels of the measured parameters within the CRC group between the obese and non-obese patients (Fig. 3), MEG3 levels were noticed to be profoundly lower in obese CRC compared to non-obese CRC patients (*P* < 0.0001). Subsequently, a substantial elevation of miR-27a expression levels was recorded in the sera of obese CRC patients compared to levels in the non-obese CRC subgroup (*P* = 0.0001). Notably, obese CRC patients showcased markedly lower IGF1 levels (*P* < 0.0001) and considerably higher IGFBP3 levels (*P* = 0.0018) than levels in the non-obese CRC subgroup. Regarding miR-181a/SIRT1, there was a non-significant difference in these parameters when compared between obese and non-obese CRC patients (*P* > 0.05). To note, the fold change for MEG3, miR-27a, and miR-181a was calculated in obese and non-obese CRC patients with normalization against their corresponding obese and non-obese controls, respectively.

### Serum MEG3 and related biomolecules have the potential for diagnosis of obesity-related CRC

**Figure 4:**
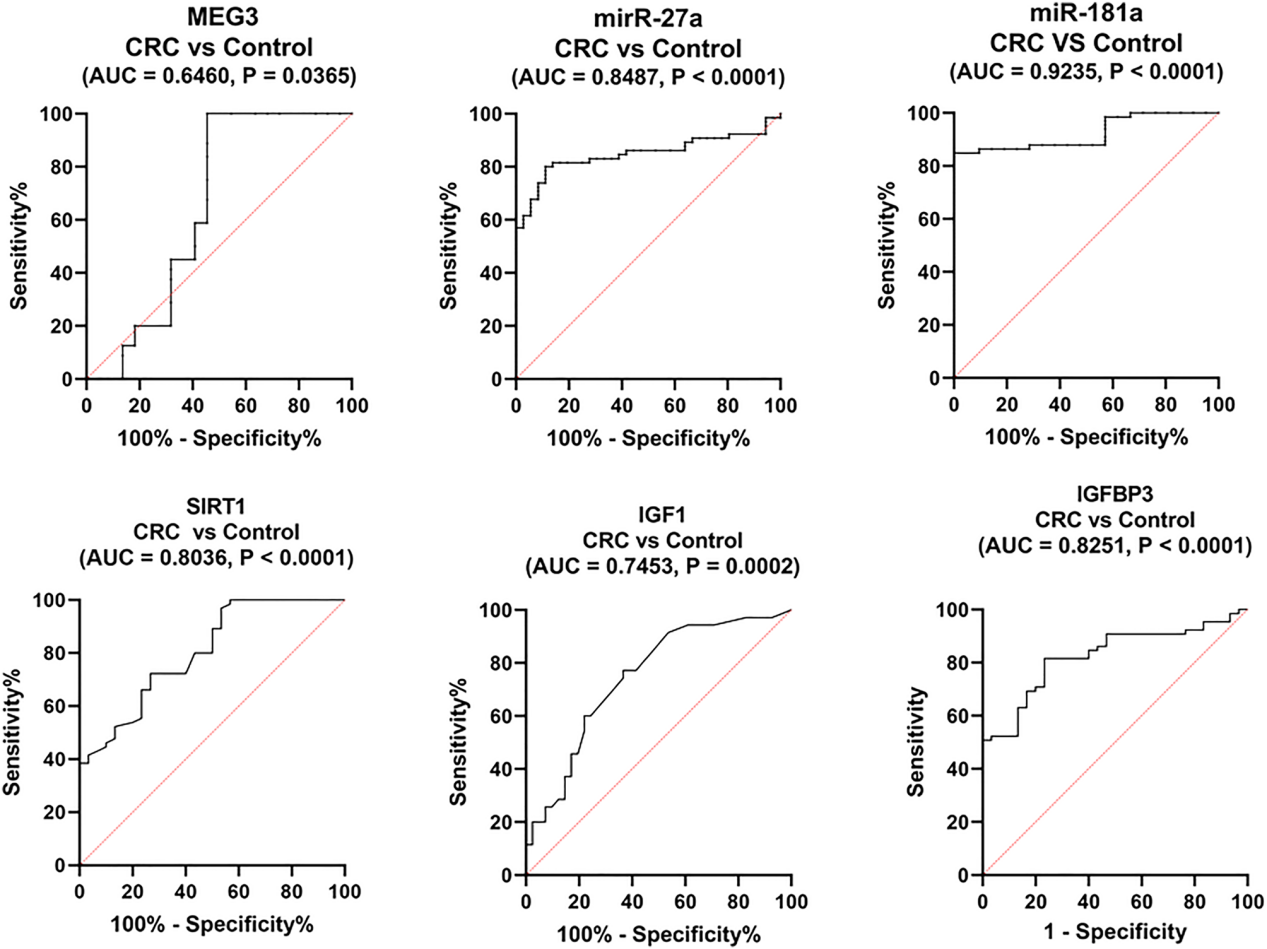
Diagnostic performance of serum MEG3, miR-27a, miR-181a, SIRT1, IGF1, and IGFBP3 in CRC. An analysis of the ROC curves for the studied parameters to distinguish between CRC patients (n = 130) and healthy controls (n = 120). *MEG3* maternally expressed gene 3, *IGF1* insulin-like growth factor 1, *IGFBP3* IGF binding protein 3, *SIRT1* sirtuin 1.

First, the potential of studied parameters in CRC diagnosis (CRC versus controls) was investigated. ROC curve analysis unveiled serum miR-181a as an excellent discriminator (AUC = 0.9235) and miR-27a, SIRT1, IGF1, and IGFBP3 levels as promising discriminators (AUC = 0.8487, 0.8036, 0.7452, and 0.8251), whereas serum MEG3 levels was only a significant discriminator (AUC = 0.646) between CRC patients and healthy controls (Fig. 4). By comparison, miR-181a showed the highest diagnostic accuracy, whereas miR-27a, SIRT1, and IGFBP3 have comparable AUCs and they were superior to IGF1, whilst MEG3 was hardly diagnostic.

Second, ROC analysis was performed using dichotomized data of obese CRC patients and their corresponding obese controls to show the diagnostic ability of the tested parameters for obesity-related CRC. Interestingly, the AUCs for MEG3, miR-27a, IGF1, and IGFBP3 were improved and recorded at 0.717, 0.975, 0.898, and 0.929, respectively, while were comparable to that for the whole CRC group for miR-181a and SIRT1 (AUC = 0.915 and 0.818, respectively) (Table 7). Notably, miR-27a and IGFBP3 were excellent discriminators, with miR-27a recorded the highest diagnostic accuracy for obesity-related CRC.

**Table 7:** Diagnostic performance of studied biomarkers.

Parameter	AUC	*P-value*	Cut-off	SN (%)	SP (%)	PPV (%)	NPV (%)
CRC vs healthy controls							
MEG3	0.646	**0.0365**	< 0.979	75	54.55	64.47	67.34
miR-27a	0.8487	** < 0.0001**	> 2.03	80	88.89	88.89	80.45
miR-181a	0.9235	** < 0.0001**	> 2.977	84.85	100	100	85.71
SIRT1	0.8036	** < 0.0001**	> 523	72.31	73.33	74.6	65.18
IGF1	0.7452	**0.0002**	< 158.1	77.14	63.41	69.44	71.7
IGFBP3	0.8251	** < 0.0001**	> 2192	81.54	76.67	79.1	79.31
Obese CRC vs obese controls							
MEG3	0.717	**0.008**	< 0.94	92.68	66.67	89.28	88.1
miR-27a	0.975	** < 0.0001**	> 1.718	95.12	88.89	91.78	94.33
miR-181a	0.915	** < 0.0001**	> 2.977	83.02	100	100	82.35
SIRT1	0.818	**0.0007**	> 424	100	53.85	72.91	100
IGF1	0.898	** < 0.0001**	< 178.6	92.68	72.22	80.25	88.88
IGFBP3	0.929	** < 0.0001**	> 2192	95.12	83.33	88.16	94
CRC obese vs non-obese							
MEG3	0.897	** < 0.0001**	< 0.675	81.58	93.1	93.44	81.16
miR-27a	0.7137	**0.002**	> 7.8	74.42	54.55	65.82	64.7
IGF1	0.9379	** < 0.0001**	< 179.9	92.31	76.92	82.27	90.19
IGFBP3	0.6643	0.002	> 4348	56.67	67.8	67.8	57.75

The best cut-off value (fold or ng/ml) was selected as the level at which the sum of sensitivity and specificity is maximum. CRC, n = 130; healthy controls, n = 120; obese CRC, n = 70; obese controls, n = 56, non-obese CRC, n = 60. *P* < 0.05 was set as the statistical significance level.

Significance values are given in bold.

*SN* sensitivity, *SP* specificity, *PPV* positive predictive value, *NPV* negative predictive value, *MEG3* maternally expressed gene 3, *IGF1* insulin-like growth factor 1, *IGFBP3* IGF binding protein 3, *SIRT1* sirtuin 1.

**Figure 5:**
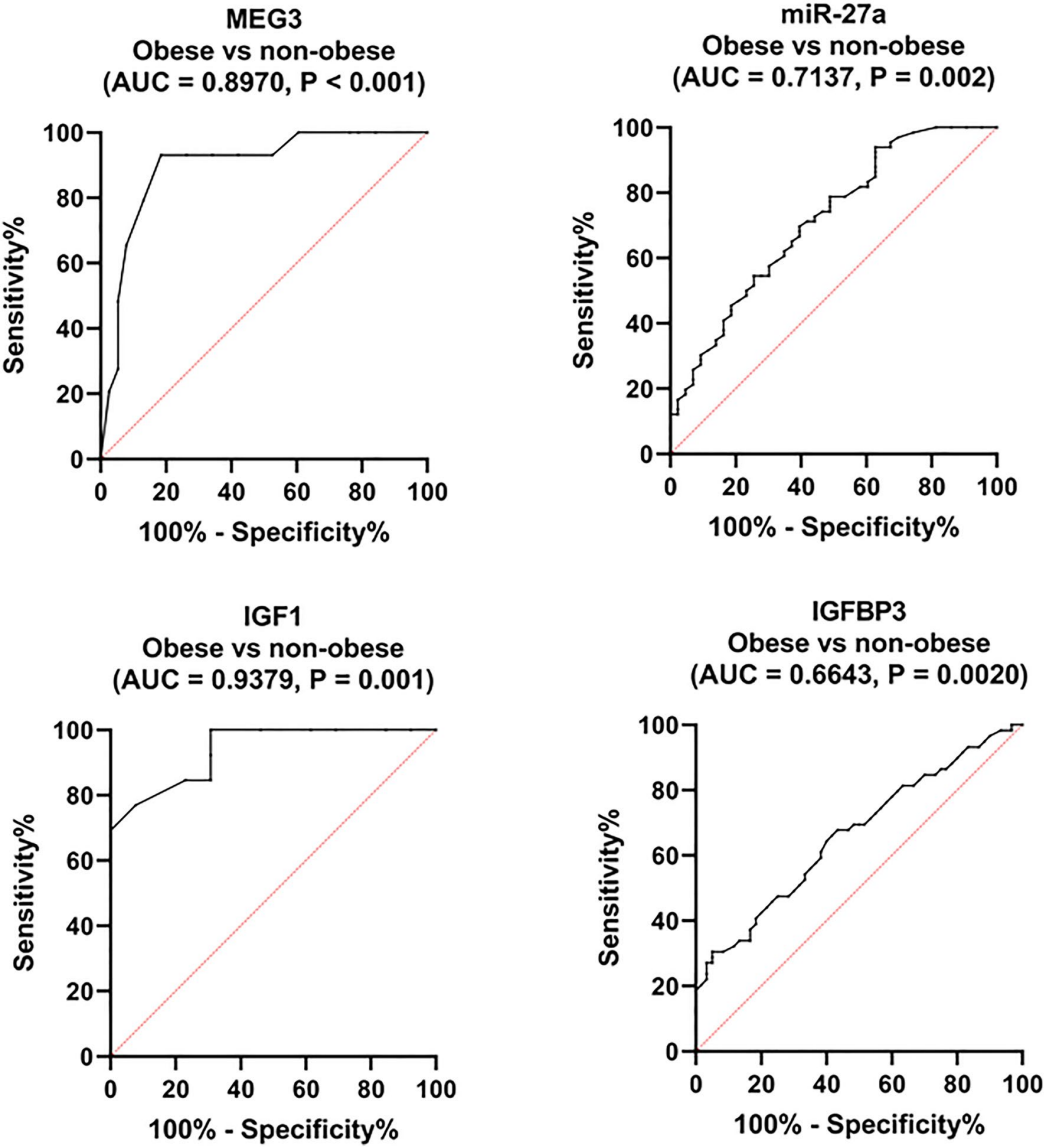
Performance of serum MEG3, miR-27a, IGF1, and IGFBP3 in discriminating obese CRC patients. An analysis of the ROC curves for the studied parameters to distinguish between obese (n = 70) and non-obese (n = 60) CRC patients. *MEG3* maternally expressed gene 3, *IGF1* insulin-like growth factor 1, *IGFBP3* IGF binding protein 3.

Third, we further assessed the discriminating ability of differentially expressed parameters between obese and non-obese CRC patients. ROC curve analysis revealed serum IGF1 as a major discriminator (AUC = 0.9379). MEG3 and miR-27a levels were promising discriminators (AUC = 0.897 and 0.7137), whereas serum IGFBP3 levels were only a significant discriminator (AUC = 0.6643) (Fig. 5). Comparison between AUCs revealed serum IGF1 > MEG3 > miR-27a > IGFBP3. The sensitivities, specificities, and positive and negative predictive values at the best cut-off values are presented in Table 7.

### miR-27a is associated with the risk of CRC in obese subjects in multivariate logistic regression analysis

Subsequently, we proceeded to configure predictor variables of the risk of obesity-related CRC in obese control subjects. We conducted univariate and then multivariate logistic regression analyses using serum levels of the differentially expressed markers (MEG3, miR-27a, IGF1, and IGFBP3) between obese and non-obese CRC patients. Despite that serum levels of the four tested variables were significantly associated with the risk of obesity-related CRC in the univariate regression analysis, multivariate analysis adjusted with confounders (age and sex) unraveled serum miR-27a as the only significant variable associated with the risk of obesity-related CRC in this study (Table 8).

**Table 8:** Association of measured parameters with obesity-related CRC compared with obese controls using logistic regression analysis.

Parameter	Beta coefficient	SE	*P-value*	OR	95% CI
Univariate analysis					
MEG3	− 2.039	0.496	**0.000**	0.13	0.049–0.344
miR-27a	1.028	0.332	**0.002**	2.79	1.457–5.366
IGF1	− 0.048	0.012	**0.0001**	0.953	0.931–0.976
IGFBP3	0.0017	0.0003	**0.000**	1.0017	1.001–1.0024
Multivariate analysis^a^					
MEG3	− 2.165	1.503	0.149	0.115	0.006–2.182
miR-27a	1.719	0.832	**0.038**	5.582	1.092–28.524
IGF1	− 0.050	0.043	0.242	0.951	0.875–1.034
IGFBP3	0.0018	0.001	0.055	1.0018	0.999–1.0037
Constant	0.482				

Univariate logistic regression analysis was performed using obese CRC cases, n = 70 cases and obese controls, n = 56. Significant variables were then entered into a stepwise-forward multivariate logistic regression analysis model with *P* < 0.05 for entering and *P* < 0.1 for removal from the model. *X*^2^ of the model = 102.82*, P* = 0.000. *P* < 0.05 was set as the statistical significance level.

Significance values are given in bold.

^a^Controlled by age and sex as covariates.

*CI* confidence interval, *OR* odds ratio, *MEG3* maternally expressed gene 3, *IGF1* insulin-like growth factor 1, *IGFBP3* IGF binding protein 3.

### Correlation study

**Figure 6:**
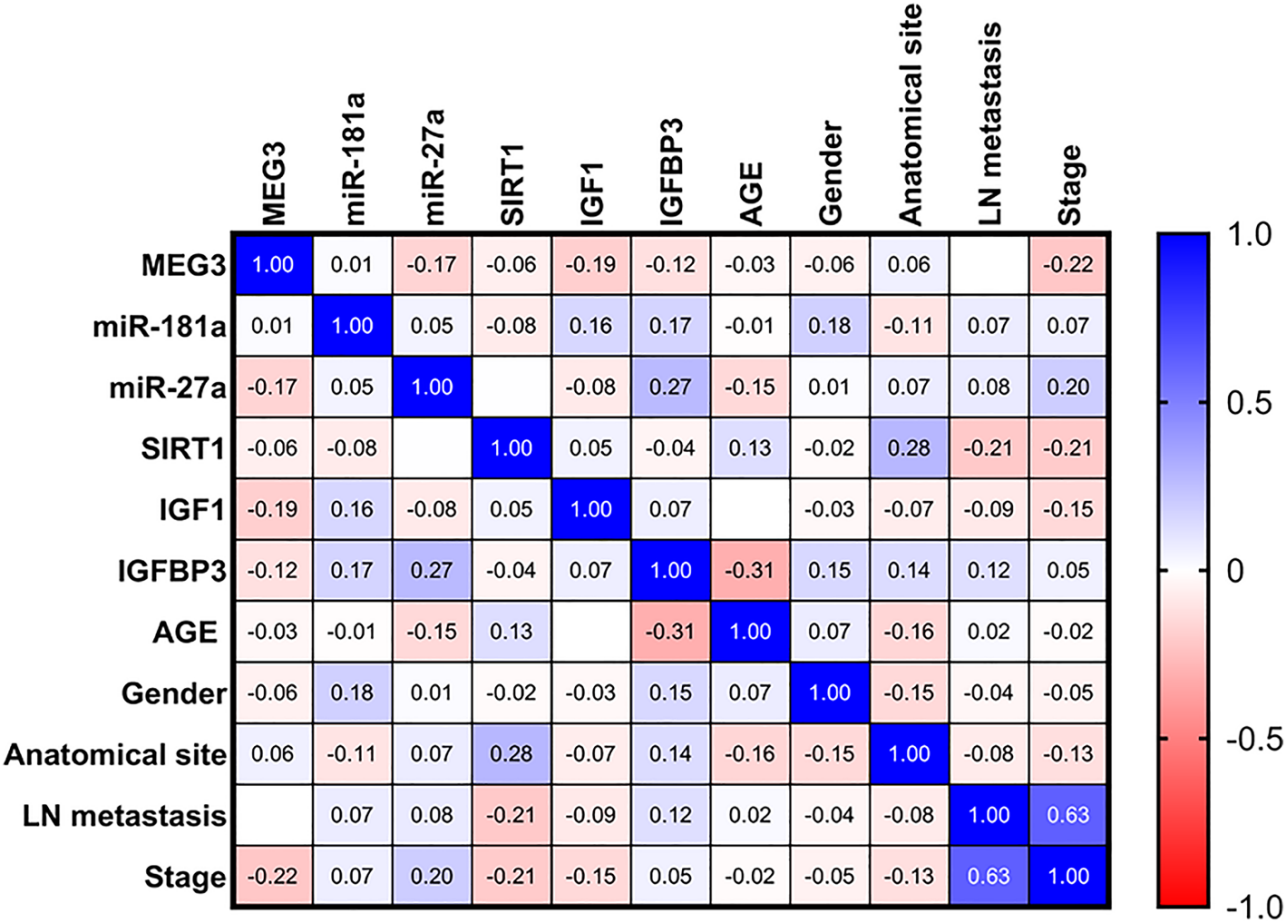
Correlations between studied parameters and clinicopathological data in CRC patients. A blue-red (cold-hot) scale was used to display the correlations in the correlation map. The blue color indicates a correlation close to 1; the red color indicates a correlation close to -1, while the white color indicates a correlation close to 0. Spearman rank coefficient was employed. *MEG3* maternally expressed gene 3, *IGF1* insulin-like growth factor 1, *IGFBP3* IGF binding protein 3, *SIRT1* sirtuin 1.

**Figure 7:**
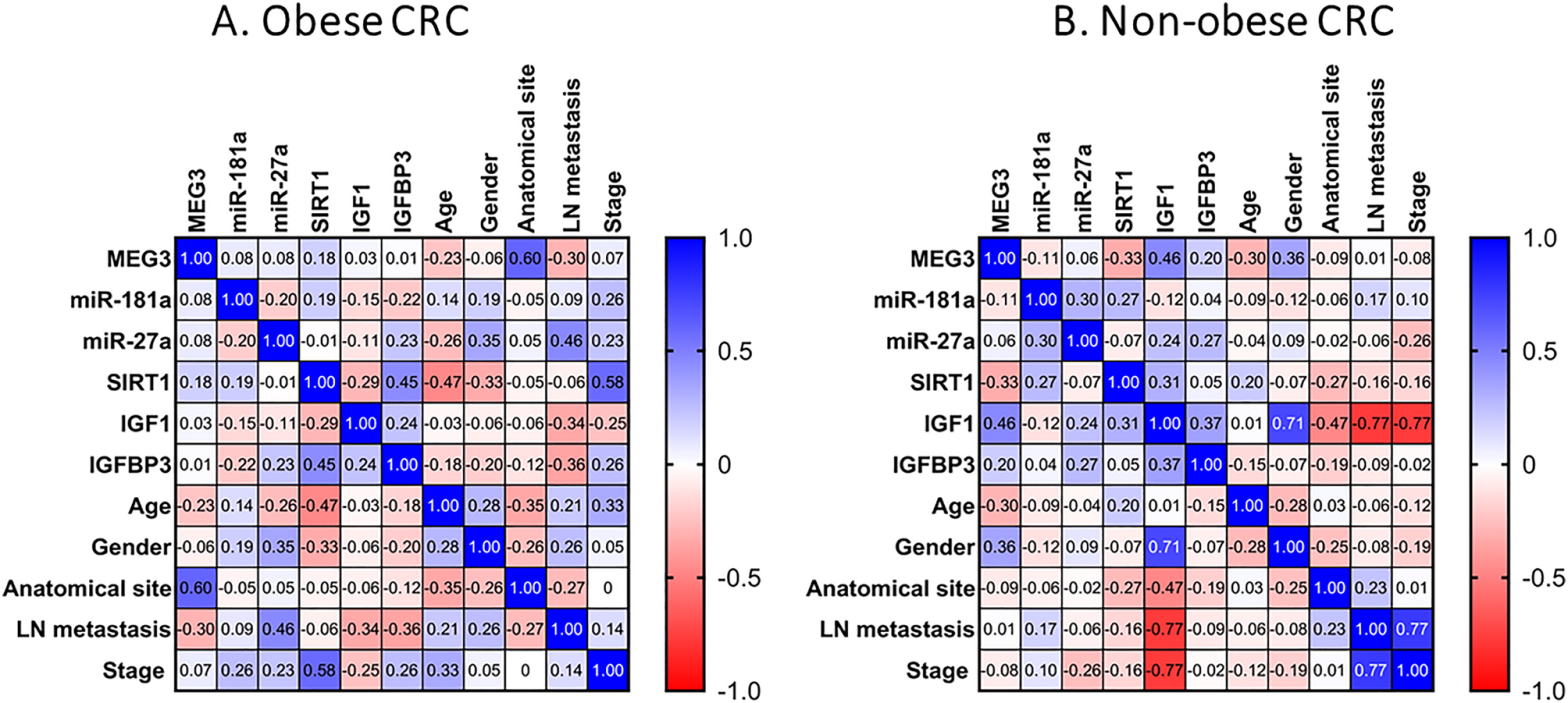
Correlations between studied parameters with each other and with demographic and clinical data in obese and non-obese CRC patients. A blue-red (cold-hot) scale was used to display the correlations in the correlation map. The blue color indicates a correlation close to 1; the red color indicates a correlation close to -1, while the white color indicates a correlation close to 0. Spearman rank coefficient was employed. *MEG3* maternally expressed gene 3, *IGF1* insulin-like growth factor 1, *IGFBP3* IGF binding protein 3, *SIRT1* sirtuin 1.

This study appraised the correlations of serum levels of studied parameters with each other and with the clinicopathological data in the whole CRC group (Fig. 6) and in separate obese and non-obese CRC patients (Fig. 7). Among the overall CRC patients, serum miR-27a expression level was positively correlated with IGFBP3 (r = 0.27, *P* = 0.029). An inverse correlation between serum IGFBP3 levels and age was evident (r = − 0.31, *P* = 0.012). Intriguingly, serum MEG3 expression level was negatively correlated with tumor stage (r = − 0.224, *P* = 0.049), while there was a positive correlation between serum SIRT1 levels and the anatomical site (r = 0.28, *P* = 0.023).

When CRC patients were dichotomized according to their obesity status additional correlations were noted (Fig. 7). In obese CRC patients, serum MEG3 was strongly and positively correlated with anatomical site (r = 0.605, *P* = 0.0001), serum miR-27a was positively correlated with LN metastasis (r = 0.46, *P* = 0.012), serum SIRT1 was positively correlated with IGFBP3 (r = 0.45, *P* = 0.013) and tumor stage (r = 0.584, *P* = 0.036), while serum IGFBP3 was negatively correlated with LN metastasis (r = − 0.36, *P* = 0.042). A positive correlation between miR-27a and miR-181a expression levels was evident in the non-obese CRC patients (r = 0.296, *P* = 0.024). Like in the overall CRC group, the correlation between miR-27a and IGFBP3 was evident in the non-obese CRC group (r = 0.27, *P* = 0.039). Interestingly, serum IGF1 was strongly correlated positively with male gender (r = 0.714, *P* = 0.01) and negatively with LN metastasis and tumor stage (r = − 0.77, *P* = 0.006 for each) among the non-obese CRC patients.

### Results of the bioinformatics analysis

**Figure 8:**
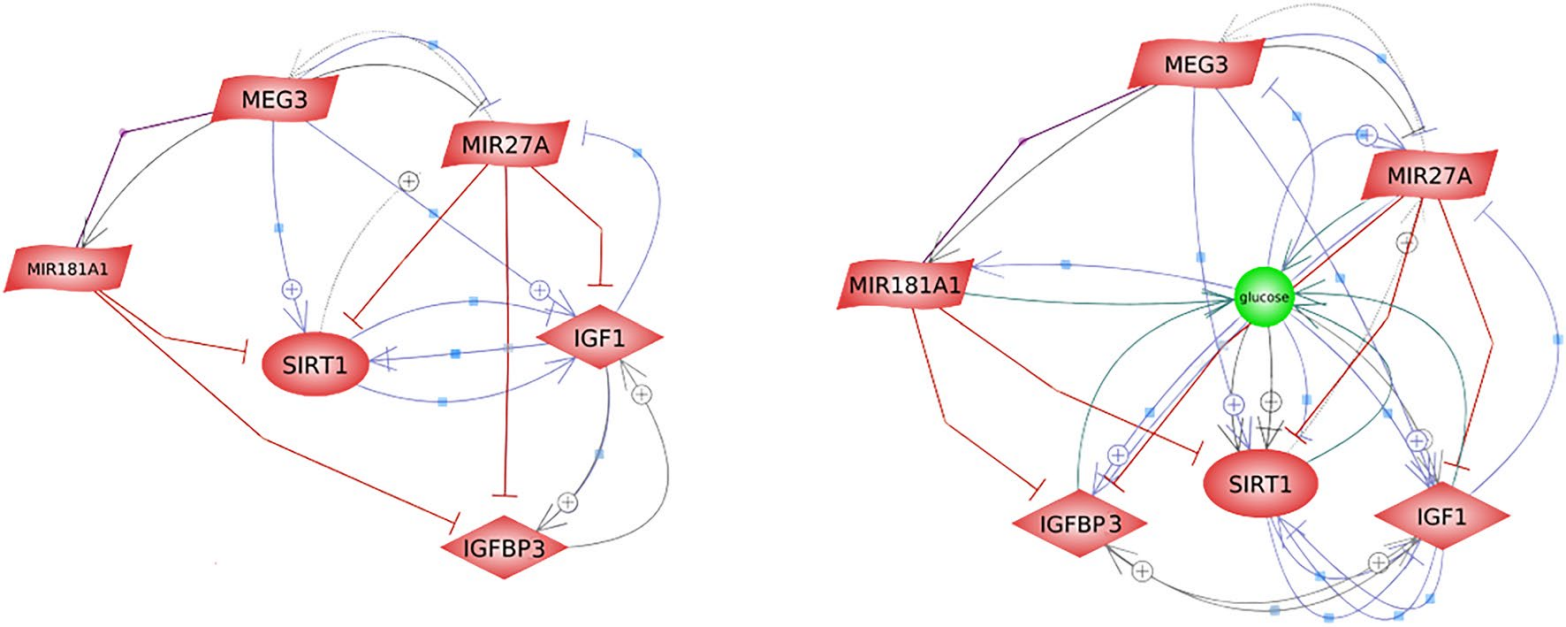
Construction of MEG3 co-expression network linked to glucose using bioinformatics. The interactions were constructed using the Pathway Studio online software. *MEG3* maternally expressed gene 3, *IGF1* insulin-like growth factor 1, *IGFBP3* IGF binding protein 3, *SIRT1* sirtuin 1.

The molecular interactions of MEG3 and selected downstream targets with each other and with glucose are visualized in Fig. 8. Based on preceding databases, the figure constructed using the Pathway Studio online tool portrays the integrated network between the measured parameters and their interrelation to glucose as a key molecule in insulin resistance, obesity, and metabolic reprogramming, which confirms our hypothesis. The network also confirms the correlations found between the measured parameters in this study as shown in Figs. 6 and 7.

## Discussion

The identification of novel screening tools is imperative to boost the early detection and treatment of CRC. Despite remarkable discoveries in the genetic predisposition of CRC, the greater part of its heritability is still missing and awaits identification. Ample evidence explicitly embraces the profound influence of SNPs in lncRNA genes on CRC risk to explain its heritability and identify robust genetic biomarkers for CRC screening and diagnosis^20,48^. To the best of our knowledge, this study is the first to emphasize the genetic association of MEG3 rs941576 with CRC susceptibility, risk factors, and clinical features and to explore circulating MEG3 expression as a valuable marker of obesity-related CRC. Interestingly, this SNP was associated with CRC risk in stratified age and gender groups and with LN metastasis and tumor stage among CRC patients, suggesting that this SNP may play a role in CRC pathogenesis and could be potentially implemented as a novel genetic marker of CRC screening, risk stratification, diagnosis, and prognosis. However, opposing our hypothesis, this SNP was not associated with either the risk of CRC in obese subjects or the expression of serum MEG3 and its related biomolecules in this study.

Evidence of the association of MEG3 genetic variants with CRC risk was unraveled in previous research^17,49,50^. The MEG3 rs7158663 variant is the most interesting polymorphic locus located on the MEG3 transcript^17^. Interestingly, this rs7158663 polymorphism was associated with CRC risk and serum MEG3 expression in Egyptian patients^50^. Mechanistically, the rs7158663 polymorphism changes the local RNA folding structure and affects miRNA:lncRNA interactions, which in turn affect the miRNA and/or MEG3 expression level^50,51^. Indeed, the rs7158663 A allele contributed to the binding of miR-4307 and miR-1265 to MEG3^17^.

This prior evidence motivated us to investigate the less-known MEG3 rs941576 as a genetic risk factor for CRC. The results of this study portrayed the association of the rs941576 minor G risk allele and minor homozygous GG genotype with heightened CRC risk in Egyptian patients. Interguingly, the risk stratification analysis unveiled the association of the GG and AG + GG genotypes with CRC risk in males and older age (≥ 50 years) group, respectively. Among CRC patients, associations of the GG and AG + GG genotypes with late tumor stages and lymph node metastasis were recorded, respectively. Intriguingly, we also observed a trend of interaction for this SNP with distant metastasis and tumor stage among obese CRC patients compared to non-obese patients. Together, these preliminary findings delineate the relationship of this SNP with CRC tumorigenesis, risk, and prognosis.

These observations recapitulate the findings of a prior study which reported the association of the MEG3 rs941576 AG + GG genotype with disease-free survival in breast cancer patients^19^. In addition, two Egyptian studies have also uncovered the association of rs941576 polymorphism with acute ischemic stroke and rheumatoid arthritis risk^38,39^. While the G allele and GG genotype of this SNP was regarded as risk factors for acute ischemic stroke^38^, the major A allele was associated with rheumatoid arthritis risk^39^. The later study reported the mechanistic impact of rs941576 in rheumatoid arthritis; the AA genotype carriers exhibited a significantly decreased serum MEG3 expression and BAX levels and increased hypoxia-inducible factor-1α and vascular endothelial growth factor levels^39^. Howbeit, we failed to configure the association of this SNP with the expression of MEG3 or its downstream targets in this study. Together, these results will stimulate further research at the cellular level. Nevertheless, MEG rs941576 is considered a novel SNP in CRC and could be predictive of CRC susceptibility and useful in risk stratification. The SNP correlations with the clinicopathological features (tumor stage and metastasis) among overall CRC and obese-CRC patients spotlight its prognostic usefulness in the clinical setting.

In this study, we further proceeded to evaluate the expression pattern and clinical relevance of circulating MEG3 in CRC and its ability to predict obesity-related CRC. Circulating MEG3 expression exhibited downregulation in CRC patients mirroring the results of prior studies in serum^49,52^, cell lines^21–23,52^, and CRC tumor tissues^45,52^. These results could be attributed to genomic deletion or abnormal methylation in the promoter of the MEG3 gene which leads to its downregulation in tumor cells^53^. Interestingly, serum MEG3 expression level showed a negative correlation with tumor stage in the current study. This result coincides with previously reported by Yin et al. that low MEG3 expression positively correlated with low histological grade, deep invasion, and advanced TNM stage in CRC tissues^54^. Together, these results confirm the role of MEG3 in CRC development and prognosis.

A more interesting and novel result is the observation of profoundly reduced levels of MEG3 expression in sera of obese CRC patients compared to their non-obese counterparts. MEG3 also potentially discriminated obese CRC from their corresponding healthy obese subjects and also from non-obese CRC patients. Serum MEG3 was also strongly correlated with the tumor anatomical site among obese CRC patients. These observations suggested that MEG3 could potentially serve as a valuable playmaker or novel therapeutic target for obesity-related CRC. This result could be explained on the basis that MEG3 has been reported to play a crucial role in tumor metabolic alterations and reprogrammed metabolic networks^55^. Tumor metabolic reprogramming is one of the hallmarks of cancer and is a key driver of obesity-associated cancer development and progression^56^. Indeed, MEG3 activated by vitamin D triggered the ubiquitin-dependent c-Myc degradation to inhibit aerobic glycolysis in CRC cells by suppressing the expression of the glycolysis-related c-Myc target genes^45^. Furthermore, MEG3 sponges miR-361-5p to promote the expression of succinate dehydrogenase and thereby, succinate accumulation in primary cells of oral lichen planus^57^.

Consequently, our hypothesis posited the plausible clinical value of MEG3 downstream targets that regulate metabolic reprogramming in obesity-related CRC. First, the expression pattern and clinical correlations of miR-27a/IGF1/IGFBP3 were examined in overall CRC as well as obese and non-obese CRC patients. This study highlighted an upregulation of serum miR-27a concomitant with MEG3 downregulation in CRC patients. This result is consistent with the notion that miR-27a is a putative MEG3 target^24^ and agrees with previous reports of upregulated miR-27a in CRC cell lines or serum and tumor tissues of CRC patients compared to controls^58,59^. miR-27a was also correlated with CRC clinical parameters in prior studies^59–61^. Intriguingly, we highlighted substantially higher levels of serum miR-27a in obese CRC versus non-obese CRC patients reflecting the role of miR-27a in the pathogenesis of obesity-related CRC. Notably, miR-27a predicted the risk of obesity-related CRC among obese subjects without CRC in multivariate analysis. Similarly, plasma miR-27a was differentially expressed in metabolic syndrome patients^62,63^. Conversely, miR-27a was downregulated in obesity^64^. Together, these results pinpoint the precious role played by miR-27a in the initiation and diagnosis of obesity-related CRC.

These results could be attributed to the paramount importance of miR-27a and its multiple downstream targets as key molecular aspects in insulin resistance and obesity^27–32^. miR-27a is also a master regulator of metabolic reprogramming in cancer cells via regulating AMP-activated protein kinase and mammalian target of rapamycin signaling pathways in CRC patients and cell lines^65^. In addition, miR-27a could affect adipocyte and cancer cell metabolism by regulating its target IGF1^17,32,66^, based on the major role of the insulin/IGF system in inhibiting apoptosis and enhancing CRC cell proliferation, differentiation, and chemoresistance^67,68^. Indeed, the mechanistic influence of MEG3/miR-27a/IGF1 axis was described in a prior study on periodontitis^66^. Together, these results explain the observed correlation of serum miR-27a with LN metastasis in obese CRC patients.

Here, this study records the downregulation of serum IGF1 and upregulation of its major binding protein IGFBP3 in CRC patients and this dysregulation was similarly reflected in the sera of obese CRC patients compared to their corresponding non-obese patients. Intriguingly, increased serum miR-27a level was correlated with elevated levels of IGFBP3 in overall CRC patients, suggesting their concordant expression in CRC. Although there is a consensus of increased serum IGF1 and reduced IGFBP3 levels in CRC patients, thus increasing IGF1 availability and its mitogenic power^67–69^, a similar Egyptian report demonstrated reduced levels of serum IGF1 in overall CRC patients and average weight and overweight/obese CRC patients compared with their corresponding controls^36^. Similar to the present results, this later study revealed IGF1 as a negative variable associated with the risk of CRC in overweight/obese patients in the univariate analysis^36^. Howbeit, it was hard to reproduce this result in the multivariate analysis, which was not analyzed in the later study. Nevertheless, the observed correlations of serum IGF1 with gender, LN metastasis, and tumor stage in non-obese CRC patients spotlight the role of the IGF system in risk stratification and pathology of obesity-CRC association.

Here, although IGFBP3 seemed to be a predictor of obesity-CRC risk in the univariate analysis, only a marginal association of IGFBP3 (*P* = 0.055) was inferred from the multivariate analysis. The recorded negative correlation between IGFBP3 and patient age further implicates IGFBP3 in CRC risk and prognosis. Particularly, the negative correlation of serum IGFBP3 with LN metastasis in obese CRC patients implicates IGFBP3 in the pathogenesis and progression of obesity-related CRC. The inconsistent results regarding the level of the IGF1 system in CRC patients may be attributed to ethnic differences, intra-individual variations, and other confounding factors. Nevertheless, the current study results configure the potential clinical utility of MEG3/miR-27a/IGF1/IGFBP3 axis, especially miR-27a in screening and early diagnosis of obesity-associated CRC.

miR-181a was depicted as putative MEG3 target in gastric cancer and multiple myeloma^25,26,70^. The role of miR-181a/SIRT1 was evident in insulin-resistant hepatocytes; miR-181a inhibits SIRT1 expression by directly binding the 3′ UTR of SIRT1 mRNA and its overexpression attenuated hepatic insulin signaling^34^. Furthermore, the impeccable role of this axis in oxidative stress was also noted in animal models^71^. Subsequently, the expression pattern and clinical relevance of circulating miR-181a/SIRT1 axis in total, obese, and non-obese CRC patients were analyzed. An upregulation of serum miR-181a and SIRT1 protein level was observed in overall CRC patients and they discriminated them from healthy controls, confirming them as surrogate biomarkers. Despite the inconsistent results about miR-181a and SIRT1 in cancer and their dual functions, as promoters and inhibitors, in certain tumors^72–76^, the results inferred from the current study mirror similar reports of heightened levels of miR-181a and SIRT1 in CRC tumor tissue and cell lines or serum^72,74,77^. To note, we observed a positive correlation of serum SIRT1 protein level with the anatomical site in overall CRC patients. Indeed, serum SIRT1 protein level was elevated in Egyptian CRC patients and previously correlated with tumor stage^77^. Previous associations of tumor tissue SIRT1 expression with the depth of tumor invasion, differentiation, tumor size, tumor tissue type, lymph node metastasis, Duke's stage, and patient age in CRC also exist^74^.

Contrary to a prior report^13^, miR-181a/SIRT1 levels weren’t significantly altered between obese and non-obese CRC patients in the current study. This controversy may be due to different samples used (serum, tissues or cell lines), different normalization controls, regulatory mechanisms, and confounding factors. Nevertheless, the correlations found between SIRT1 and IGFBP3 and between SIRT1 and tumor stage in obese CRC patients bolster its possible role in the progression of obesity-related CRC. The remarked positive correlation between serum SIRT1 and IGFBP3 among obese CRC patients could be explained on the basis that an interaction exists between SIRT1, IGFBP3, and IGF-1/phosphoinositide 3-kinase (PI3K)/protein kinase B (AKT) signal transduction and seems to play pleiotropic effects in malignancy^78^. This correlation and that observed between miR-181a and miR-27a among non-obese CRC patients potentially suggests that these markers interplay in the pathogenesis of obesity-related CRC, which requires further evaluation at the cellular level. Nevertheless, our bioinformatics analysis findings portrayed the possible interactions of MEG3 and its studied downstream targets with each other and with glucose (Fig. 8) that confirms their relation to insulin resistance, obesity, and metabolic reprogramming.

Nonetheless, it is imperative to acknowledge certain limitations that constrain the findings of the current research. Firstly, the construction of appropriate cell lines is imperative to validate the studied SNP and related axes. Secondly, although the sample size was of sufficient power, it is necessary to include larger cohort in future clinical investigations. In addition, the clinical samples were collected from one hospital; thus, future multicenter studies are warranted. Ultimately, in the context of precision medicine, the identification of suitable biomarkers and therapeutic targets for the early detection and therapy of obesity-related CRC is of paramount importance, albeit a challenging endeavor.

## Conclusion

This study is the first to provide preliminary evidence of the genetic association of MEG3 rs941576 with CRC susceptibility, risk factors, and clinical features. The results also accentuate circulating MEG3/miR-27a/IGF1/IGFBP3 as valuable markers of the early detection of obesity-related CRC, with miR-27a is associated with its risk. This axis along with SIRT1 correlates with tumor parameters and could have clinical utility in obesity-related CRC prognosis. The current study advocates that the addition of such tools in clinical practice may facilitate timely implementation of prevention strategies, improve patient counseling, screening, and individualized testing, and add to the therapeutic repertoire of CRC.

### Supplementary Information

Supplementary Information.

## Data Availability

All data generated or analyzed during this study are included in this published article and its supplementary file.

## Abbreviations

CI: Confidence interval
CRC: Colorectal cancer
ESR: Erythrocyte sedimentation rate
GAPDH: Glyceraldehyde 3-phosphate dehydrogenase
GWAS: Genome-wide association studies
HWE: Hardy–Weinberg equilibrium
IBD: Inflammatory bowel disease
IGF1: Insulin-like growth factor 1
IGFBP3: IGF binding protein 3
LN: Lymph node
lncRNA: Long non-coding RNA
MEG3: Maternally expressed gene 3
miRNA: MicroRNA
OR: Odds ratio
ROC: Receiver-operating characteristic
SIRT1: Sirtuin 1
SNP: Single nucleotide polymorphism
