# Supplementary material for: Association of lncRNA MEG3 rs941576 polymorphism, expression profile, and its related targets with the risk of obesity-related colorectal cancer: potential clinical insights
Source: Sci Rep. 2024 May 4;14:10271. doi: 10.1038/s41598-024-60265-6 (PMC11069513; doi:10.1038/s41598-024-60265-6)
Supplement: Supplementary file 1 — Supplementary Information. [file 41598_2024_60265_MOESM1_ESM.docx]

**Supplementary Information**

**Table S1: MEG 3 rs941576 (A/G) MAF in the controls**

|  | **MAF in the control group** | **Global MAF**  **(Ensembl release Jan 2024)** | **Highest population MAF (Ensembl release Jan 2024)** |
| --- | --- | --- | --- |
| MEG 3 rs941576 | G = 0.12 | G = 0.38 | G= 0.49 |

**Table S2**: **Hardy-Weinberg equilibrium for rs941576 (A/G) in control and CRC groups**

| **rs941576 A/G exact test for Hardy-Weinberg equilibrium (n=250)** | | | | | | |
| --- | --- | --- | --- | --- | --- | --- |
|  | **AA** | **AG** | **GG** | **A** | **G** | **P-value** |
| **Control** | 95 | 21 | 4 | 211 | 29 | 0.067 |
| **CRC** | 94 | 19 | 17 | 207 | 53 | **<0.0001** |

**Table S3 Association of MEG3 rs941576 A/G SNP with anatomical site in CRC patients**

| **rs941576 A/G association with anatomical site** | | | | | | | |
| --- | --- | --- | --- | --- | --- | --- | --- |
| **Model** | **Genotype** | **Colon**  **(n=79)** | **Rectum**  **(n-51)** | **OR^a^ (95% CI)** | **P-value** | **AIC** | **BIC** |
| Codominant | A/A | 62 (78.5%) | 32 (62.8%) | 1.00 | 0.27 | 172.4 | 189.6 |
|  | A/G | 10 (12.7%) | 9 (17.6%) | 1.39 (0.49-3.94) |  |  |  |
|  | G/G | 7 (8.9%) | 10 (19.6%) | 2.44 (0.80-7.38) |  |  |  |
| Dominant | A/A | 62 (78.5%) | 32 (62.8%) | 1.00 | 0.16 | 171.1 | 185.4 |
|  | A/G-G/G | 17 (21.5%) | 19 (37.2%) | 1.80 (0.79-4.10) |  |  |  |
| Recessive | A/A-A/G | 72 (91.1%) | 41 (80.4%) | 1.00 | 0.13 | 170.8 | 185.2 |
|  | G/G | 7 (8.9%) | 10 (19.6%) | 2.28 (0.77-6.80) |  |  |  |
| Overdominant | A/A-G/G | 69 (87.3%) | 42 (82.3%) | 1.00 | 0.74 | 173 | 187.3 |
|  | A/G | 10 (12.7%) | 9 (17.6%) | 1.19 (0.43-3.30) |  |  |  |
| Log-additive | --- | --- | --- | 1.53 (0.91-2.58) | 0.11 | 170.5 | 184.8 |

SNPStats online software was employed. ^a^ adjusted with age, sex, and obesity in a logistic regression model. Statistical significance was set as *P*<0.05. AIC, Akaike Information Criterion; BIC, Bayesian Information Criterion; CI, confidence interval; CRC, colorectal cancer; OR, odds ratio.

**Table S4 Association of MEG3 rs941576 A/G SNP with lymph node metastasis in CRC patients**

| **rs941576 A/G association with LN metastasis** | | | | | | | |
| --- | --- | --- | --- | --- | --- | --- | --- |
| **Model** | **Genotype** | **No LN metastasis**  (n=70) | **LN metastasis**  (n=60) | **OR^a^ (95% CI)** | **P-value** | **AIC** | **BIC** |
| Codominant | A/A | 54 (77.1%) | 40 (66.7%) | 1.00 | 0.078 | 176 | 193.2 |
|  | A/G | 8 (11.4%) | 11 (18.3%) | 2.77 (0.94-8.18) |  |  |  |
|  | G/G | 8 (11.4%) | 9 (15%) | 2.51 (0.80-7.87) |  |  |  |
| Dominant | A/A | 54 (77.1%) | 40 (66.7%) | 1.00 | 0.024 | 174 | 188.3 |
|  | A/G-G/G | 16 (22.9%) | 20 (33.3%) | **2.65 (1.11-6.31)** |  |  |  |
| Recessive | A/A-A/G | 62 (88.6%) | 51 (85%) | 1.00 | 0.21 | 177.5 | 191.8 |
|  | G/G | 8 (11.4%) | 9 (15%) | 2.01 (0.67-6.01) |  |  |  |
| Overdominant | A/A-G/G | 62 (88.6%) | 49 (81.7%) | 1.00 | 0.11 | 176.5 | 190.9 |
|  | A/G | 8 (11.4%) | 11 (18.3%) | 2.31 (0.81-6.58) |  |  |  |
| Log-additive | --- | --- | --- | **1.73 (1.01-2.99)** | 0.043 | 175 | 189.3 |

SNPStats online software was employed. ^a^ adjusted with age, sex, and obesity in a logistic regression model. Bold indicates statistical significance, *P*<0.05. AIC, Akaike Information Criterion; BIC, Bayesian Information Criterion; CI, confidence interval; CRC, colorectal cancer; LN, lymph node; OR, odds ratio.

**Table S5 Association of MEG3 rs941576 A/G SNP with distant metastasis in CRC patients**

| **rs941576 A/G association with distant metastasis** | | | | | | |  |
| --- | --- | --- | --- | --- | --- | --- | --- |
| **Model** | **Genotype** | **No Distant metastasis**  (n=110) | **Distant metastasis**  (n=20) | **OR^a^ (95% CI)** | **P-value** | **AIC** | **BIC** |
| Codominant | A/A | 78 (70.9%) | 16 (80%) | 1.00 | 0.71 | 120.2 | 137.4 |
|  | A/G | 17 (15.4%) | 2 (10%) | 0.55 (0.11-2.69) |  |  |  |
|  | G/G | 15 (13.6%) | 2 (10%) | 0.73 (0.14-3.66) |  |  |  |
| Dominant | A/A | 78 (70.9%) | 16 (80%) | 1.00 | 0.43 | 118.3 | 132.6 |
|  | A/G-G/G | 32 (29.1%) | 4 (20%) | 0.62 (0.19-2.09) |  |  |  |
| Recessive | A/A-A/G | 95 (86.4%) | 18 (90%) | 1.00 | 0.78 | 118.8 | 133.2 |
|  | G/G | 15 (13.6%) | 2 (10%) | 0.80 (0.16-3.98) |  |  |  |
| Overdominant | A/A-G/G | 93 (84.5%) | 18 (90%) | 1.00 | 0.47 | 118.4 | 132.7 |
|  | A/G | 17 (15.4%) | 2 (10%) | 0.57 (0.12-2.78) |  |  |  |
| Log-additive | --- | --- | --- | 0.78 (0.36-1.71) | 0.52 | 118.5 | 132.8 |

SNPStats online software was employed. ^a^ adjusted with age, sex, and obesity in a logistic regression model. Statistical significance was set as *P*<0.05. AIC, Akaike Information Criterion; BIC, Bayesian Information Criterion; CI, confidence interval; CRC, colorectal cancer; OR, odds ratio.

**Table S6 Association of MEG3 rs941576 A/G SNP with tumor stage in CRC patients**

| \| **rs941576 A/G association with tumor Stage** \| \| \| \| \| \| \| \| \| --- \| --- \| --- \| --- \| --- \| --- \| --- \| --- \| \| **Model** \| **Genotype** \| **Stage I-II**  (n=86) \| **Stage III-IV**  (n=44) \| **OR^a^ (95% CI)** \| **P-value** \| **AIC** \| **BIC** \| \| Codominant \| A/A \| 66 (76.7%) \| 28 (63.6%) \| 1.00 \| 0.11 \| 166 \| 183.2 \| \| A/G \| 12 (13.9%) \| 7 (15.9%) \| 1.31 (0.44-3.90) \| \| G/G \| 8 (9.3%) \| 9 (20.4%) \| **3.34 (1.07-10.38)** \| \| Dominant \| A/A \| 66 (76.7%) \| 28 (63.6%) \| 1.00 \| 0.1 \| 165.8 \| 180.1 \| \| A/G-G/G \| 20 (23.3%) \| 16 (36.4%) \| 2.03 (0.87-4.75) \| \| Recessive \| A/A-A/G \| 78 (90.7%) \| 35 (79.5%) \| 1.00 \| 0.041 \| 164.3 \| 178.6 \| \| G/G \| 8 (9.3%) \| 9 (20.4%) \| **3.16 (1.04-9.61)** \| \| Overdominant \| A/A-G/G \| 74 (86%) \| 37 (84.1%) \| 1.00 \| 0.93 \| 168.4 \| 182.8 \| \| A/G \| 12 (13.9%) \| 7 (15.9%) \| 1.05 (0.36-3.03) \| \| Log-additive \| --- \| --- \| --- \| **1.73 (1.01-2.97)** \| 0.044 \| 164.4 \| 178.7 \| |
| --- | --- | --- | --- | --- | --- | --- | --- | --- | --- | --- | --- | --- | --- | --- | --- | --- | --- | --- | --- | --- | --- | --- | --- | --- | --- | --- | --- | --- | --- | --- | --- | --- | --- | --- | --- | --- | --- | --- | --- | --- | --- | --- | --- | --- | --- | --- | --- | --- | --- | --- | --- | --- | --- | --- | --- | --- | --- | --- | --- | --- | --- | --- | --- | --- | --- | --- | --- | --- | --- | --- | --- | --- | --- | --- | --- | --- |

SNPStats online software was employed. ^a^ adjusted with age, sex, and obesity in a logistic regression model. Bold indicates statistical significance, *P*<0.05. AIC, Akaike Information Criterion; BIC, Bayesian Information Criterion; CI, confidence interval; CRC, colorectal cancer; OR, odds ratio.


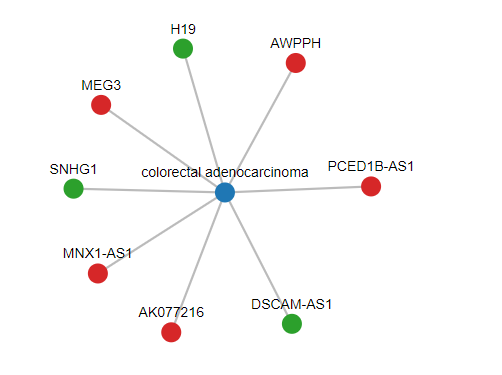


**Figure S1. TOP lncRNAs linked to colorectal carcinoma.** Data was generated from the LncRNADisease v3.0.
